# ushr: Understanding suppression of HIV in R

**DOI:** 10.1186/s12859-020-3389-x

**Published:** 2020-02-11

**Authors:** Sinead E Morris, Luise Dziobek-Garrett, Andrew J Yates, in collaboration with the EPIICAL Consortium

**Affiliations:** Department of Pathology and Cell Biology, Columbia University Medical Center, New York, NY, USA

**Keywords:** HIV, ART, Biphasic decay, Mathematical model, R package

## Abstract

**Background:**

HIV/AIDS is responsible for the deaths of one million people every year. Although mathematical modeling has provided many insights into the dynamics of HIV infection, there is still a lack of accessible tools for researchers unfamiliar with modeling techniques to apply them to their own clinical data.

**Results:**

Here we present ushr, a free and open-source R package that models the decline of HIV during antiretroviral treatment (ART) using a popular mathematical framework. ushr can be applied to longitudinal data of viral load measurements, and provides processing tools to prepare it for computational analysis. By mathematically fitting the data, important biological parameters can then be estimated, including the lifespans of short and long-lived infected cells, and the time to reach viral suppression below a defined detection threshold. The package also provides visualization and summary tools for fast assessment of model results.

**Conclusions:**

ushr enables researchers without a strong mathematical or computational background to model the dynamics of HIV using longitudinal clinical data. Increasing accessibility to such methods may facilitate quantitative analysis across a broader range of independent studies, so that greater insights on HIV infection and treatment dynamics may be gained.

## Background

In 2017, one million people died from HIV/AIDS globally, including 50,000 children less than one year old [1, 2]. Mathematical modeling has provided many insights into the dynamics of HIV infection in chronically-infected adults undergoing antiretroviral treatment (ART). In particular, simple frameworks describing temporal changes in viral load have identified distinct subsets of infected cells (such as CD4 T cells) that decay at different rates and differentially impact infection dynamics [3–8]. Such models accurately capture the classic ‘biphasic’ pattern of viral decline: the rapid loss of short-lived infected cells drives an initial sharp decrease in viral load, and the loss of other long-lived infected populations drives a subsequent slower decline phase [3, 4, 9]. Fitting biphasic models to clinical data revealed that over 90% of infected cells in chronically-infected adults are short-lived, with an average lifespan of one day [4]. This highlighted the highly active landscape of HIV infection.

Despite extensive characterization of infected cell populations in certain cohorts, there is a need for greater accessibility to computational tools, so that researchers unfamiliar with mathematical modeling can apply these techniques to their own clinical data. Consistent biphasic patterns of viral decay have been demonstrated in very young infants undergoing ART, similar to those described in adults [10]. Thus the biphasic model can be applied across different age groups and may provide important insights into HIV infection and treatment dynamics, if made accessible to the wider clinical community.

Through our package ushr (understanding suppression of HIV in R), we provide tools for modeling the dynamics of HIV infection in a cohort of individuals undergoing ART. Using the canonical biphasic model of viral decay, the package estimates the lifespans of short and long-lived infected cell populations, and the time at which individuals achieve viral suppression. We also provide visualization and summary tools for fast assessment of model results. More generally, we present ushr as an accessible framework to encourage analysis and compilation of HIV dynamics across a broader range of independent studies. This will enhance our understanding of HIV treatment effects by providing robust, quantitative inferences of individual parameters that can be compared among different cohorts.

## Implementation

### Data preparation

Raw clinical data is often unsuitable for mathematical analysis of viral decline, and eventual suppression, during ART. For example, observations may be noisy and sparse; some individuals may reach suppression and others may not; and some may not experience consistent decline of viral load, due to factors such as drug resistance or poor adherence to treatment. Therefore, prior to any analysis, data must be processed to exclude individual trajectories that cannot be appropriately modeled.

In ushr, we only consider individuals who reach suppression within a particular timeframe (specified by the user). By default, suppression is defined as having at least one viral load measurement below a specific detection threshold, *d*. However, we also allow the user to define suppression as sustaining at least two consecutive measurements below *d*. All measurements below the detection threshold are set to *d*/2, in line with previous work [11]. To isolate the kinetics leading to initial suppression, viral load trajectories are truncated after the first measurement below *d*.

To differentiate ‘true’ decay dynamics from cases of viral rebound (due to factors such as drug resistance or poor treatment adherence), we isolate the viral load data that form a consistent decreasing sequence towards suppression, such that each measurement is within a pre-defined range of the previous measurement. This buffer range ensures that brief increases in viral load arising from noise and measurement error do not exclude individuals from the analysis. Although the buffer could, in theory, permit gradual growth in viral load over time, such dynamics are unlikely to arise in individuals transitioning towards suppression. Finally, we allow initial increases in viral load (for example, arising from pharmacological delays in drug action) by defining the beginning of each decreasing sequence as the maximum observation from the first *n* measurements, where *n* will depend on the data under consideration and can be specified by the user.

In addition to filtering and processing existing data according to the above inclusion criteria, ushr also provides functionality to simulate noisy data from the underlying mathematical model. We use such data below to validate the fitting procedure.

### Mathematical model

To model HIV decline during ART, ushr leverages previously developed ordinary differential equations (ODEs) that describe interactions between the virus and its target cells, primarily CD4 T cells (see, for example, refs. [4–8] and Additional file 1). If ART completely blocks virus replication, and the clearance of cell-free virus occurs on a faster timescale than the lifetime of infected cells, the course of viral load during treatment, *V*(*t*), can be expressed as

1 $$ V(t) = A\exp\left(-\delta t\right) + B\exp\left(- \gamma t\right).  $$

Here, *δ* and *γ* are the death rates of short and long-lived productively infected target cells, respectively; *A*+*B* is the viral load at ART initiation (i.e. *V*(*t*=0)); and *A*/(*A*+*B*) is the proportion of infected cells at ART initiation that are short-lived (Additional file 1) [6]. Adopting standard practice, we omit the dynamics of latently infected cells and assume their contribution to viral load is minimal relative to short and long-lived infected cells [4]. We also assume cells produce infectious virus immediately upon infection. If we instead incorporated a delay before productive infection, *δ* and *γ* would reflect the loss rates of short-lived productively infected cells and long-lived non-productively infected cells, respectively [12].

Equation 1 is referred to as the biphasic model: viral load initially decays rapidly, reflecting the loss of short-lived infected cells (at rate *δ*), and then enters a second, slower decline phase reflecting the loss of long-lived infected cells (at rate *γ*). This pattern has been observed in both adults and children during ART [4, 6, 10]. Alternatively, for subjects exhibiting only one decline phase (for example, due to sparse or delayed observations), one can employ a single phase version of Eq. 1 given by

2 $$ V(t) = \hat{B}\exp\left(- \hat{\gamma} t\right),  $$

where the decay rate $\hat {\gamma }$ can reflect the fast or the slow phase of HIV decay.

It is important to highlight that while the above equations are traditionally applied to ART containing reverse-transcriptase inhibitors (RTIs) and protease inhibitors (PIs), treatments including integrase inhibitors (IIs) are also becoming increasingly common. Under II therapy, viral trajectories exhibit three phases of exponential decline that reflect (1) the loss of short-lived productively infected cells; (1b) the loss of short-lived non-productively infected cells; and (2) the loss of long-lived non-productively infected cells [12]. In order to fit such trajectories, we also include a triphasic exponential model given by

3 $$ V(t) = A\exp\left(-\delta t\right) + A_{b}\exp(-\delta_{b} t) + B\exp(- \gamma t),  $$

where *δ* and *γ* represent the loss of short-lived productively infected cells and the loss of long-lived non-productively infected cells, respectively (phases (1) and (2) described above). These parameters have the same interpretation as in the biphasic model with delay to productive infection. The additional decay rate, *δ*_*b*_, represents the loss of short-lived non-productively infected cells (phase (1b)), which is only observed under II therapy. For the remainder of the paper we focus mainly on the biphasic and single phase models for RTI/PIs; further information on implementing the triphasic model for IIs may be found in the package documentation.

### Time to suppression

In addition to modeling the course of HIV decline, we also estimate the time to reach virologic suppression (‘time to suppression’ (TTS)) below a defined threshold using both parametric and nonparametric methods. For the parametric approach, TTS is calculated as the time at which *V*(*t*)=*x*, where *x* is the suppression threshold, and *V*(*t*) is given by Eq. 1 for the biphasic model, Eq. 2 for the single phase model, and Eq. 3 for the triphasic model. In contrast, the nonparametric approach does not require a mathematical model. Instead, we apply linear interpolation between the first measurement below the suppression threshold and the preceding measurement. Time to suppression is then defined as the time at which the interpolation line crosses the suppression threshold. Estimates can also be quoted as the time since ART initiation, if this date is recorded.

### Model fitting

We obtain independent parameter estimates, with 95% confidence intervals, for each subject by fitting either the biphasic, single phase, or triphasic model to the corresponding viral load data using maximum likelihood optimization (as described previously [13]). We recommend the biphasic and single phase models for individuals undergoing RTI/PI-based therapy, and the triphasic model for II-based therapy. Data are log_10_-transformed prior to fitting and optimization is performed using optim() in R [14], assuming normally-distributed errors. After fitting, we use the resulting parameter estimates to calculate the infected cell lifespans (for example in the biphasic model, 1/*δ* and 1/*γ* for short and long-lived infected cells, respectively).

Only subjects with a minimum number of measurements above the detection threshold (to be specified by the user) are fit using each model. We recommend at least nine observations for the triphasic model, six for the biphasic model, and three for the single phase model. Subjects with fewer than the required number of measurements are not included in the model fitting procedure, although they can still be included in nonparametric TTS calculations.

Finally, in the case of RTI/PIs, some individuals may have large differences in viral load between the first and second measurements. This is common with sparse clinical data and suggests an unobserved transition from the fast to the slow decay phase. To prevent such occurrences biasing the estimated slope of decay when fitting the single phase model, we remove the first measurement if the difference in viral load is greater than a specified threshold.

## Results

### ushr captures dynamics of previously published data

We first demonstrate the general capability of ushr by using the package to analyze publicly available data from the ACTG 315 clinical trial. These data have been described previously [5, 15, 16], and are available with the package, or at https://sph.uth.edu/divisions/biostatistics/wu/datasets/ACTG315LongitudinalDataViralLoad.htm(accessed 15 September 2019). Briefly, longitudinal measurements of HIV viral load are documented for 46 chronically-infected adults undergoing RTI/PI-based ART. The assay detection threshold was 100 RNA copies ml ^−1^, and data were recorded up to 28 weeks following treatment initiation.

**Fig. 1:**
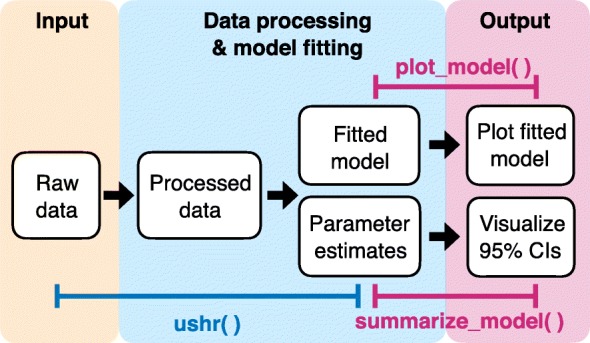
Schematic of package pipeline. Raw input data is first processed to isolate and prepare individual trajectories that adhere to the specific inclusion criteria. The processed data are then fit using the biphasic or single phase model to obtain fitted model predictions and corresponding parameter estimates. These first two steps can be achieved in one line of code using the ushr() function (or ushr_triphasic() if fitting the triphasic model).The output information can then be visualized; for example by plotting the model fits, using plot_model(), and inspecting the 95% parameter confidence intervals (CIs) using summarize_model()

Given such a dataset, one can use ushr to process the data and fit the applicable mathematical model (here, biphasic or single phase). First, individual trajectories that adhere to the specific inclusion criteria are identified i.e. those that form a consistent decreasing sequence towards the detection threshold, and include the minimum number of observations required for model fitting (six for the biphasic model and three for the single phase model). Our algorithm then fits the appropriate model to the data and computes the best-fit parameter estimates with 95% confidence intervals. Note that the single phase model is only applied to individuals ineligible for the biphasic model. Both the data processing and model fitting steps can be achieved in one line of code using the ushr() function (or ushr_triphasic() if fitting the triphasic model). The resulting model trajectories can be visualized using plot_model() (Fig 1 and Additional file 2).

**Fig. 2:**
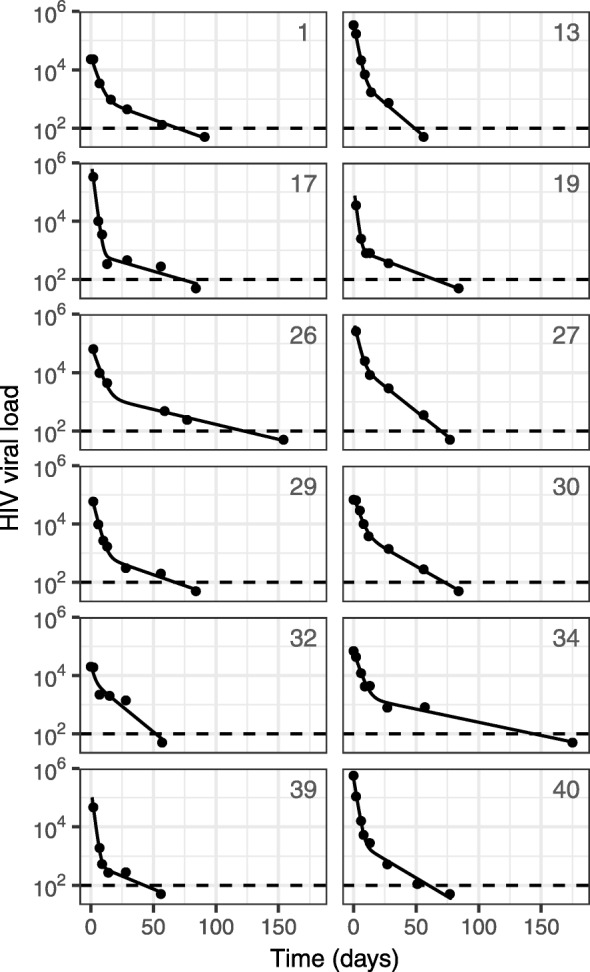
Biphasic model fit to published data. Twelve subjects from the ACTG 315 study were fit using the biphasic model. The solid lines are the model predictions, points are the observed data, and the dashed lines represent the experiment detection threshold. Subject identifiers are included in the top right corner of each panel. Units of HIV viral load are RNA copies ml ^−1^

**Fig. 3:**
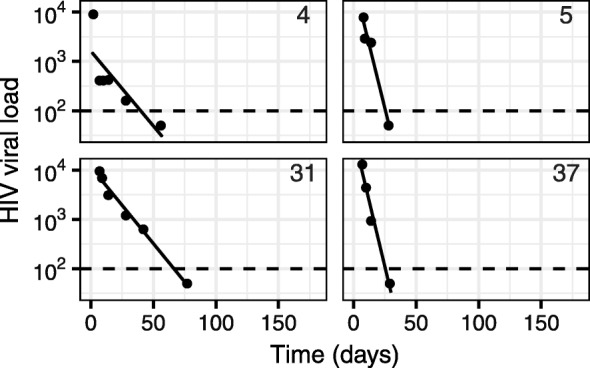
Single phase model fit to published data. Four subjects from the ACTG 315 study were fit using the single phase model. The solid lines are the model predictions, points are the observed data, and the dashed lines represent the experiment detection threshold. Subject identifiers are included in the top right corner of each panel. Units of HIV viral load are RNA copies ml ^−1^

For the ACTG 315 data, twelve individuals were fit with the biphasic model (Fig 2), and four with the single phase model (Fig 3). Although some single phase subjects had sufficient data to fit the biphasic model (i.e. at least six observations), the resulting 95% parameter confidence intervals were either unattainable or sufficiently wide to indicate an unreliable fit. This can occur, for example, when one of the decay phases is poorly documented (i.e. has few data points). As a result, the subjects were re-fit with the single phase model. This re-fitting step is automated in the package, although the user can control the confidence interval width above which a biphasic fit is deemed unreliable.

A summary of the model applied to each subject, and the associated infected cell lifespans, can be obtained using summarize_model() (Additional file 2). The biphasic fits from the ACTG 315 data had median lifespans of 2.1 days (SD = 0.8) and 25.6 days (SD = 11.9) for short and long-lived infected cells, respectively. These estimates are in line with previous analyses of these data; for example, 2.3 days and 31.3 days, respectively [5]. The single phase fits had a median lifespan of 9.1 days (SD = 5.8). To assess the robustness of parameter estimates for each subject, one can view their corresponding 95% confidence intervals (Additional file 2). Pairwise parameter correlation plots can also be viewed to assess dependencies at the population-level. We present further analyses comparing parameter estimates with their true values below.

Finally, one can calculate the TTS for all eligible subjects with the get_TTS() function. The user can specify the threshold viral load under which a subject is defined to have reached suppression. Here we set the suppression threshold equal to the detection threshold. The user can also specify whether the parametric or nonparametric method should be used. For the ACTG 315 data, the median TTS estimates were 65.7 (SD = 31) and 69.8 days (SD = 37.9) for the parametric and nonparametric methods, respectively (Additional file 2).

### Parameter estimates are accurate for sufficiently high resolution data

Study sampling design can have a substantial impact on the reliability of parameter estimates obtained from model fitting. For example, short-lived cells have an average lifespan on the order of days, which may be difficult to estimate accurately in a clinical setting, given the challenge of obtaining frequent observations. Indeed, one may expect to overestimate this lifespan, as the most reliable fits will tend to derive from individuals with slower decay and thus more information regarding the first decline phase.

We investigated this possibility by comparing parameter estimates derived from simulated data with different sampling frequencies (Additional file 3). Briefly, we simulated data using the simulate_data() function in ushr, and specified the number of subjects with nsubjects = 200. This command generates individual trajectories from the biphasic model (Eq. 1), using underlying parameters that are sampled from lognormal distributions with user-specified mean and standard deviation. Gaussian noise (with zero mean and user-specified standard deviation) is added on the log_10_ scale, and the number of observed time points is randomly sampled for each subject from a pre-defined range. This approach assumes subjects are sampled at regular intervals until either the end of the study period or they are lost to follow-up.

To investigate the impact of sampling resolution, we varied the frequency of observation times using three different scenarios. In our high resolution dataset, individuals were sampled four times per month; in the medium resolution dataset this was reduced to twice per month; and in the low resolution dataset, the frequency was reduced further to once per month. In all cases, individuals were sampled for a minimum of three months and maximum of one year.

As above, we fit the high, medium, and low resolution datasets using ushr(), then calculated an average deviation score that summarized the difference between the true and estimated parameter values. For each parameter *p*, and study resolution *r*, we defined the average deviation as

4 $$ D_{p,r} = \frac{1}{n_{p,r}}\sum_{i = 1}^{n_{p,r}}\left(\theta_{i} - \hat{\theta}_{i}\right)/\theta_{i},  $$

where *n*_*p*,*r*_ is the number of subjects in the study that were fit with the biphasic model, *θ*_*i*_ is the true parameter value for subject *i*, and $\hat {\theta }_{i}$ is the estimated value. Note that for the parameters *A* and *B*, we log_10_-transformed *θ*_*i*_ and $\hat {\theta _{i}}$ prior to calculating the deviance. For TTS values, we used the parametric approach to calculate *θ*_*i*_ and $\hat {\theta }_{i}$ from the corresponding true and estimated parameters, respectively. We repeated the process for 100 different randomly sampled underlying parameter distributions, resulting in 1200 average deviation scores (4 parameters, 3 study resolutions, and 100 repetitions).

**Fig. 4:**
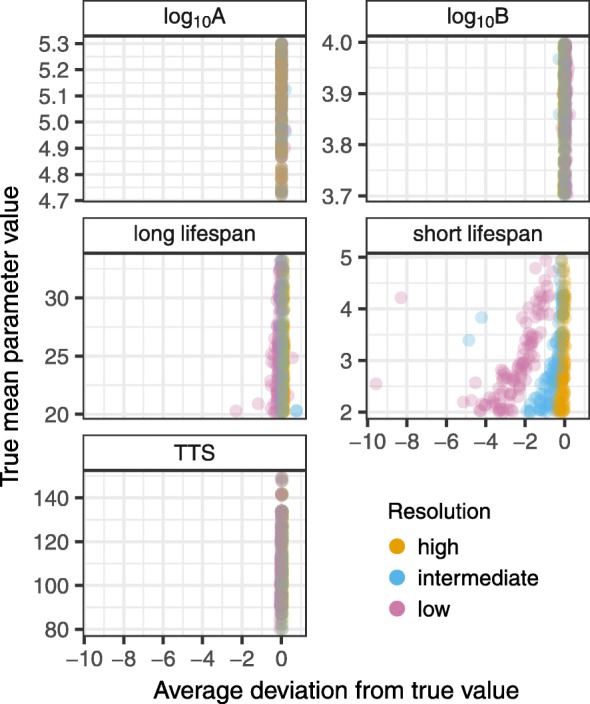
Average deviation from true parameters. Average deviation scores were calculated for each parameter and study resolution using Eq. 4. These were then compared with the true mean parameter value used to simulate each study. Simulated parameter values reflect the following units: days for TTS and the short and long lifespans of infected cells; RNA copies ml ^−1^ for *A* and *B*. TTS stands for time to suppression

In general, estimates of the short-lived lifespan from the low resolution studies had the largest deviation (Fig 4). The values were consistently negative, indicating overestimation; and the shortest lifespans exhibited the largest deviances. These results are consistent with the intuition that the fastest decay phases are most vulnerable to infrequent sampling. All other parameter values had relatively low deviation scores, although the long-lived lifespan estimates also exhibited a slight negative bias at low sampling frequencies.

**Fig. 5:**
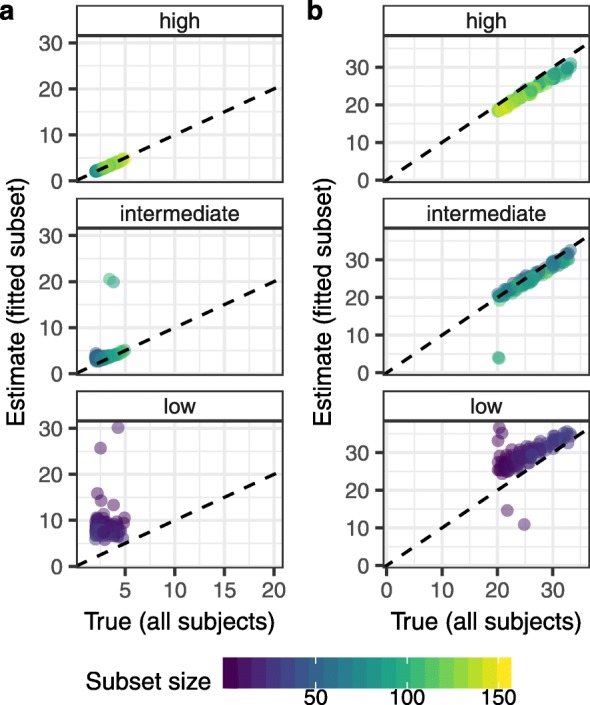
Comparison of fitted estimates to population-wide values. Median values (in days) of the short-lived (**a**) and long-lived (**b**) lifespans across all subjects in each simulated study were compared to the median estimates from the subset analyzed with the biphasic model. Low, intermediate, and high sampling resolutions are depicted in the panels. Dashed lines represent the one-to-one threshold where estimates are equal to true values

Finally, we explored the extent to which estimates from the subset of individual trajectories fit using the biphasic model could be extrapolated across the whole study population. We focussed on the short and long-lived lifespans since these exhibited the greatest deviances. For each study and lifespan, we compared the true population median to the median estimate from the fitted subset. Unsurprisingly, estimates from low resolution studies were the poorest representation of the true population-wide values (Fig 5). Both lifespans were overestimated, reflecting problems with infrequent observations, as discussed above. In addition, fewer subjects in these low resolution studies had sufficient data for fitting, leading to smaller sample sizes and a greater departure from the population average.

In contrast, estimates from the intermediate and high resolution studies were in good agreement with the true population average, although the long-lived lifespans were slightly underestimated at higher values. At intermediate and high resolutions, the effects of sample size and infrequent observations are reduced. However, our algorithm for simulating data is such that individuals with slow second decline phases are more likely to be lost to follow-up before reaching suppression. Thus our fitting may be biased towards subjects with shorter long-lived lifespans than the population average. The effect will be most pronounced when the average long-lived lifespan is high and restrictions from infrequent sampling are minimal.

Overall, these results demonstrate that our fitting procedure can reliably estimate individual parameters at intermediate and high sampling resolutions, and that these estimates represent an unbiased sample of parameters across the population. Conversely, caution must be taken when interpreting parameters at low resolutions, and in extrapolating these estimates to the wider population.

### ushr performs comparably to nonlinear mixed effects modeling

In ushr we take an individual-based approach by independently fitting data from each subject that meets our inclusion criteria and our minimum number of observations. Since the model equations are pre-defined (i.e. do not need to be specified by the user), our package has the advantage of straightforward implementation. Conversely, another common method for fitting longitudinal clinical data is to use a population-based approach such as nonlinear mixed effects (NLME) modeling [5, 17]. Briefly, the NLME framework aims to describe inter-subject variation in the population by assuming a form for the distribution of parameters at the population-level. This approach has the advantage that information from all individuals who meet the inclusion criteria can be leveraged to inform the overall fits, regardless of the number of available measurements. However, existing NLME software packages are typically general purpose, and require users to manually define model structure according to their specific need. In addition, imposing population-level distributions may cause NLME to perform poorly on individuals with outlying dynamics.

In order to compare estimates obtained from NLME modeling with those obtained above from ushr, we re-fit the low, medium, and high resolution datasets using the NLME package saemix in R (Additional files 4, 5, 6, 7, and 8) [18]. In brief, we defined the parameters *A*,*B*,*δ*, and *γ* as log-normal distributions with individual random effects, and used saemix to fit Eq. 1 and estimate the corresponding population-level distributions.

**Fig. 6:**
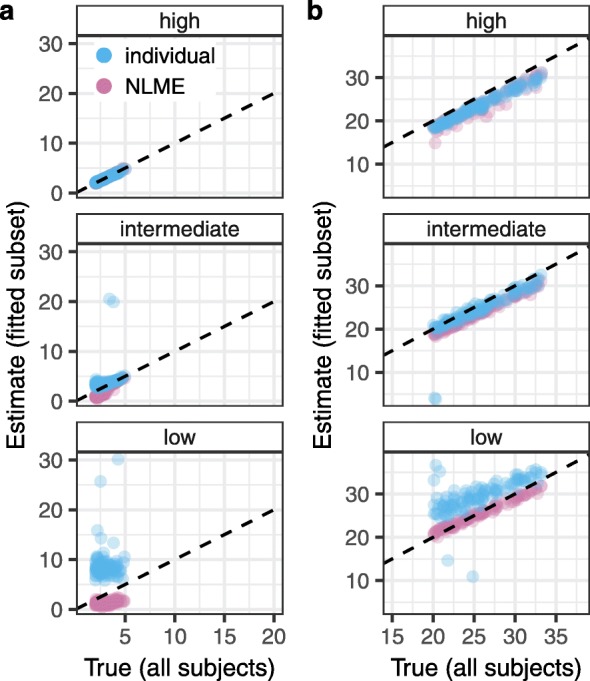
Comparison of ushr’s individual approach with nonlinear mixed effects modeling. Median values (in days) of the short-lived (**a**) and long-lived (**b**) lifespans across each study population were compared to the corresponding estimates from the subset that were fit using the individual-based approach of ushr (blue) or nonlinear mixed effects (NLME) modeling (purple). Note that the subsets fit using ushr are generally smaller than those fit using NLME due to the minimum number of observations required for individual inclusion. Low, intermediate, and high sampling resolutions are depicted in the panels. Dashed lines represent the one-to-one threshold where estimates are equal to true values

In general, we found lifespan estimates from the NLME approach were similar to, or marginally worse than, those obtained from ushr for the intermediate and high resolution data (Fig 6). Conversely, for the low resolution data, the NLME lifespans were closer to the true population average, although the short-lived lifespans were slightly underestimated. In summary, our individual-based algorithm performs comparably to the population-based NLME approach, with clear discrepancies only becoming apparent at low sampling resolutions.

## Conclusions

ushr provides free and open-source software for researchers looking to model viral decline in HIV-infected individuals undergoing ART. The package implements a mathematical framework that has been shown to capture viral dynamics in adults and children [4, 6, 10], and automates all major steps of the analysis: data processing and filtering, model fitting and parameter estimation, and output visualization. Specifics of the particular data set can be chosen by the user, such as the detection threshold of the measurement assay and the duration of the study period. Users can also specify tuning parameters of the modeling process, including the minimum number of data points required for fitting. Importantly, the package can be used for both RTI/PI and II-based therapy, although the choice of model must be guided by the data at hand, taking into consideration the type of therapy and the observed pattern of decay.

More generally, the package provides tailored and accessible tools for mathematical analysis of longitudinal HIV data. Although care must be taken when interpreting estimates from low resolution studies, and extrapolating values to larger populations, the procedure can be used to elucidate and compare virus dynamics across individuals undergoing ART. Notably, the package performs comparably to a population-based NLME approach, and has the advantage that the model is pre-defined. Software that requires user-defined equations or model frameworks may be more challenging to implement for those new to computational modeling. Thus, in developing this package we hope to encourage more quantitative analyses of HIV clinical studies, so that greater insights on viral infection and treatment dynamics can be gained.

## Availability and requirements

**Project name:** ushr**Project home page:**https://github.com/SineadMorris/ushr**Operating system(s):** Platform independent**Programming language:** R (≥3.5.3)**Other requirements:** R packages dplyr (≥0.8.0.1), tidyr (≥0.8.3), and ggplot2 (≥3.1.1)**License:** MIT licence**Any restrictions to use by non-academics:** None

## Supplementary information

**Additional file 1** Model derivation.

**Additional file 2** Example of package implementation using previously published data from the ACTG 315 clinical trial.

**Additional file 3** Code used to generate all simulated data and conduct corresponding ushr analyses.

**Additional file 4** Code used to compare ushr analyses with nonlinear mixed effects modeling at the population level.

**Additional file 5** Additional individuals fit using nonlinear mixed effects modeling.

**Additional file 6** Datasets generated during the ushr simulation study.

**Additional file 7** True parameter values generated during the ushr simulation study.

**Additional file 8** Parameter estimates obtained during the ushr simulation study.

## Data Availability

The ushr package is available on the Comprehensive R Archive Network at https://cran.r-project.org/web/packages/ushr/index.html; and on GitHub at https://github.com/SineadMorris/ushr. The ACTG 315 data are available in the package, and at https://sph.uth.edu/divisions/biostatistics/wu/datasets/ACTG315LongitudinalDataViralLoad.htm. The code to analyze these data is available in Additional file 2. The code used to generate and analyze the simulated data is available in Additional file 3. The code and data used to compare ushr analyses with nonlinear mixed effects modeling are available in Additional files 4, 5, 6, 7, and 8. Packages used during figure generation were: ggplot2, viridis, cowplot, dplyr, and tidyr [19–23].

## Abbreviations

ART: antiretroviral treatment
II: integrase inhibitor
NLME: nonlinear mixed effects
PI: protease inhibitor
RTI: reverse-transcriptase inhibitor
TTS: time to suppression
