# Supplementary material for: ushr: Understanding suppression of HIV in R
Source: BMC Bioinformatics. 2020 Feb 11;21:52. doi: 10.1186/s12859-020-3389-x (PMC7014720; doi:10.1186/s12859-020-3389-x)
Supplement: Supplementary file 1 — Additional file 1 Model derivation. [file 12859_2020_3389_MOESM1_ESM.pdf]

Additional File 1: Model derivation

Sinead E. Morris, Luise Dziobek-Garrett, Andrew J. Yates

The following ODE system describes the dynamics of HIV infection and treatment. Uninfected target cells, T, are infected by free virions at per-capita rate k to become short-lived infected cells, T^*. These cells produce virions at rate p and die at rate δ. Other cells, M, can also become infected at per-capita rate k_M, giving rise to long-lived infected cells, M^*. These long-lived infected cells also produce virions at rate p and die at rate $\gamma < \delta$. Virions can either be infectious (i.e. capable of infecting new cells), V_I, or non-infectious, V_{NI}, and decay at rate $c \gg \delta, \gamma$. Assuming a reverse-transcriptase inhibitor blocks infection of new cells with efficacy e_1 and a protease inhibitor blocks the production of infectious virions with efficacy e_2, we can write the following equations

$$\frac{dT^*}{dt} = (1 - e_1)kTV - \delta T^* \tag{1}$$
$$\frac{dM^*}{dt} = (1 - e_1)k_M MV - \gamma M^* \tag{2}$$
$$\frac{dV_I}{dt} = (1 - e_2)(pT^* + pM^*) - cV_I \tag{3}$$
$$\frac{dV_{NI}}{dt} = e_2(pT^* + pM^*) - cV_{NI}, \tag{4}$$

as outlined previously [1-4]. Note that for any $e_2 \in [0, 1]$, the total number of free virions, V, is given by

$$\begin{aligned}\frac{dV}{dt} &= \frac{dV_I}{dt} + \frac{dV_{NI}}{dt} \\ &= (1 - e_2)(pT^* + pM^*) + e_2(pT^* + pM^*) - c(V_I + V_{NI}) \\ &= pT^* + pM^* - cV.\end{aligned}$$

Thus, the efficacy of the protease inhibitor has no observable impact on the combined dynamics of infectious and non-infectious virions.

Now, assuming that viral dynamics occur on a faster timescale than infected cells, we can make the quasi-steady state assumption that $dV/dt \approx 0$, so that

$$V(t) = \tfrac{p}{c}\big(T^* + M^*\big). \tag{5}$$

Finally, if we assume reverse transcriptase inhibition is completely effective (i.e. $e_1 = 1$), we can solve Eqns 1 and 2 to give $T^* = T_0 e^{-\delta t}$ and $M^* = M_0 e^{-\gamma t}$, were T_0 and M_0 are the initial numbers of short and long-lived infected cells, respectively. Substituting these solutions into Eqn. 5 gives the final expression

$$V(t) = Ae^{-\delta t} + Be^{-\gamma t},$$

where $A = pT_0/c$ and $B = pM_0/c$. In other words, viral load should decay exponentially with an initial phase of rapid decline, reflecting the loss of short-lived infected cells (δ), and then enter a second, slower decline phase, reflecting the loss of long-lived infected cells (γ). Finally, note that $A/(A+B) = T_0/(T_0 + M_0)$ i.e. the proportion of the total initial infected cell population that is short-lived.

References

1. Perelson, A.S., Neumann, A.U., Markowitz, M., Leonard, J.M., Ho, D.D.: HIV-1 dynamics in vivo: viron clearance rate, infected cell life-span, and viral generation time. Science 271(5255), 1582–1586 (1996).
2. Wu, H., Ding, A.A.: Population HIV-1 dynamics in vivo: applicable models and inferential tools for virological data from AIDS clinical trials. Biometrics 55(2), 410–418 (1999)
3. Nowak, M.A., May, R.M.: Virus Dynamics: Mathematical Principles of Immunology and Virology. Oxford University Press, New York,USA (2000)
4. Shet, A., Nagaraja, P., Dixit, N.M.: Viral decay dynamics and mathematical modeling of treatment response: evidence of lower in vivo fitness of HIV-1 subtype C. J Acquir Immune Defic Syndr 73(3), 245–251 (2016).
