# Supplementary material for: ushr: Understanding suppression of HIV in R
Source: BMC Bioinformatics. 2020 Feb 11;21:52. doi: 10.1186/s12859-020-3389-x (PMC7014720; doi:10.1186/s12859-020-3389-x)
Supplement: Supplementary file 2 — Additional file 2 Example of package implementation using previously published data from the ACTG 315 clinical trial. [file 12859_2020_3389_MOESM2_ESM.pdf]

# Additional File 2: ushr captures dynamics of previously published data

Sinead E. Morris, Luise Dziobek-Garrett, Andrew J. Yates

This file includes all code used to model the ACTG 315 data with `ushr`. The information can also be found in the package vignette.

## Data

The data consist of 46 chronically-infected adults from the ACTG 315 clinical trial undergoing RTI/PI-based ART (i.e. anti-retroviral treatment containing two reverse-transcriptase inhibitors and one protease inhibitor). These data are available at https://sph.uth.edu/divisions/biostatistics/wu/datasets/ACTG315LongitudinalDataViralLoad.htm (date accessed: 15 September 2019), and have been described previously (see, for example, refs. 1–3 below). The data include longitudinal HIV viral load measurements up to 28 weeks following treatment initiation. The detection threshold was 100 copies/ml and observations are recorded as $\log_{10}$ RNA copies/ml.

The raw data are available through `ushr`. We begin by loading the package and printing the first six rows to identify our columns of interest: the observations ('log.10.RNA.'), the timing of these observations ('Day'), and the identifier for each subject ('Patid').

```
library(ushr)

print(head(actg315raw))
```

```
##   Obs.No Patid Day log10.RNA.    CD4
## 1      1     1   0     4.3617 221.76
## 2      2     1   2     4.3617 159.84
## 3      3     1   7     3.5315 210.60
## 4      4     1  16     2.9777 204.12
## 5      5     1  29     2.6435 172.48
## 6      6     1  57     2.1139 270.94
```

We then transform the $\log_{10}$ viral load measurements into absolute values, and rename the column headings to those required by `ushr`.

```
actg315 <- actg315raw %>%
    mutate(vl = 10^log10.RNA.) %>%
    select(id = Patid, time = Day, vl)

print(head(actg315))
```

```
##   id time         vl
## 1  1    0 22998.5259
## 2  1    2 22998.5259
## 3  1    7  3400.1651
## 4  1   16   949.9484
## 5  1   29   440.0479
```

```
## 6  1   57   129.9870
```

We can now visualize these data using the `plot_data()` function.

```
plot_data(actg315, detection_threshold = 100)
```

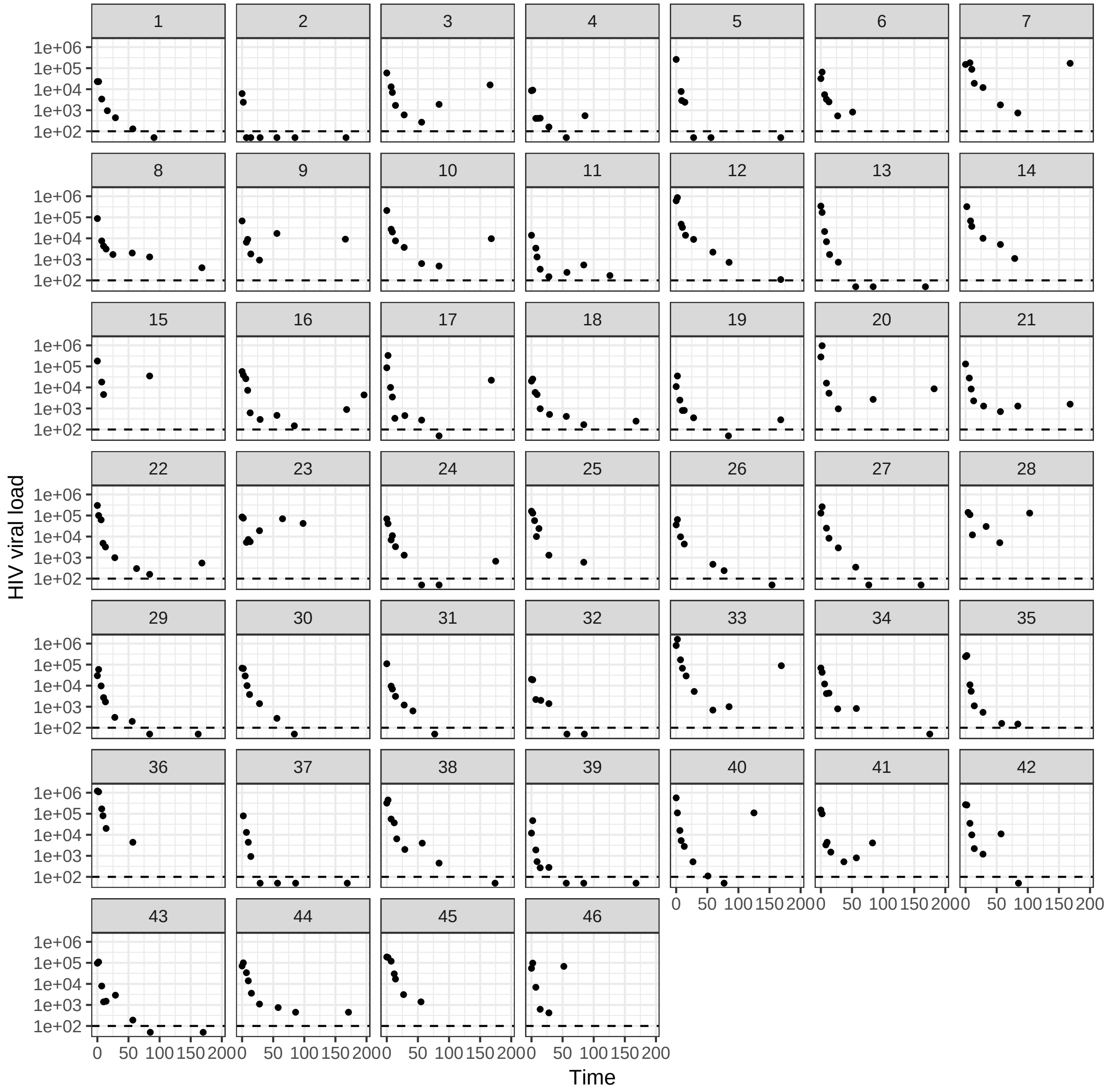


## Model fitting and output visualization

To fit the model to these data in just one line of code, we use the `ushr()` function. This processes the data to discount any individuals who do not meet the inclusion criteria, and then fits either the biphasic or single phase model to each remaining trajectory, depending on the number of available observations (see the main text for more details).

```
model_output <- ushr(data = actg315, detection_threshold = 100)
```

With the fitted model output, we can then plot both the biphasic and single phase fits as follows

```r
plot_model(model_output, type = "biphasic", detection_threshold = 100)
```

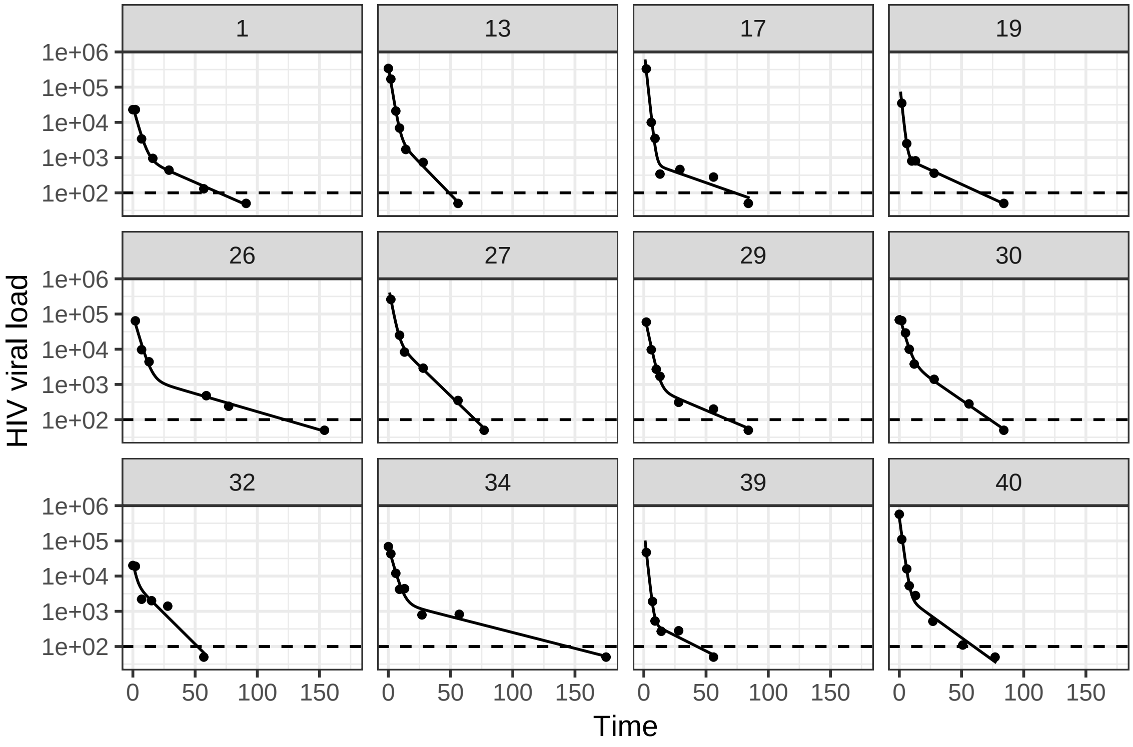


```r
plot_model(model_output, type = "single", detection_threshold = 100)
```

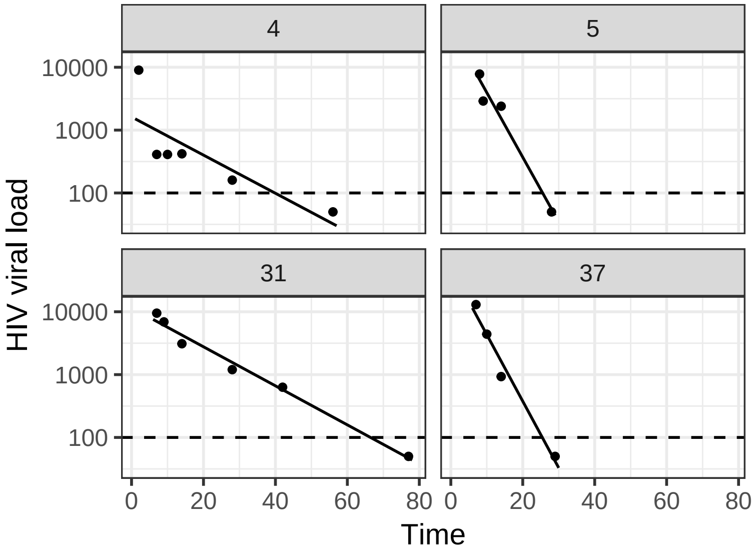


We can also visualize a summary of the fitting procedure and parameter estimates using `summarize_model()`. This creates a list with the following elements: (i) a summary of which subjects were successfully fit, with the corresponding infected cell lifespan estimates (`summary`); (ii) summary statistics for the biphasic parameter estimates (`biphasicstats`); and (iii) summary statistics for the single phase parameter estimates (`singlestats`).

```r
actg315_summary <- summarize_model(model_output, data = actg315, stats = TRUE)

head(actg315_summary$summary)
```

```
##   id Included        Model ShortLifespan LongLifespan SingleLifespan
## 1  1      Yes     Biphasic          3.25        28.08
## 2  2       No
## 3  3       No
## 4  4      Yes Single phase                                    14.3
## 5  5      Yes Single phase                                    4.22
## 6  6       No
```

```r
actg315_summary$biphasicstats
```

```
## # A tibble: 6 x 4
##   Param              Median         SD Model
##   <chr>               <dbl>      <dbl> <chr>
## 1 A             135000      367000      Biphasic
## 2 B               1890        6540      Biphasic
## 3 delta              0.482       0.185  Biphasic
## 4 gamma              0.0391      0.0227 Biphasic
## 5 LongLifespan      25.6        11.9    Biphasic
## 6 ShortLifespan      2.08        0.834  Biphasic
```

```r
actg315_summary$singlestats
```

```
## # A tibble: 3 x 4
##   Param             Median        SD Model
##   <chr>              <dbl>     <dbl> <chr>
## 1 Bhat            26900     23300     Single phase
## 2 gammahat            0.154     0.098 Single phase
## 3 SingleLifespan      9.1       5.76  Single phase
```

For a better understanding of parameter identifiability, one can also print the parameter estimates for each individual and model, along with their corresponding 95% confidence intervals. The latter are estimated from the hessian matrix obtained during optimization.

```r
head(model_output$biphasicCI)
```

```
## # A tibble: 6 x 5
##      id param    estimate     lowerCI     upperCI
##   <dbl> <chr>       <dbl>       <dbl>       <dbl>
## 1     1 A      27545.      19879.      38167.
## 2     1 delta      0.308       0.228       0.414
## 3     1 B       1174.         701.       1966.
## 4     1 gamma      0.0356      0.0283      0.0448
## 5    13 A     385392.     300226.     494717.
## 6    13 delta      0.512       0.455       0.575
```

```r
head(model_output$singleCI)
```

```
##   id    param     estimate      lowerCI      upperCI
## 1  4     Bhat 1.621519e+03 5.480429e+02 4.797663e+03
## 2  4 gammahat 6.993061e-02 3.909354e-02 1.250920e-01
## 3  5     Bhat 4.223169e+04 1.962813e+04 9.086529e+04
## 4  5 gammahat 2.368478e-01 1.952923e-01 2.872457e-01
## 5 31     Bhat 1.158218e+04 8.791109e+03 1.525937e+04
## 6 31 gammahat 7.151531e-02 6.466642e-02 7.908956e-02
```

Pairwise parameter scatter plots can also be viewed to assess dependencies at the population-level. Note that correlations between parameters may indicate underlying biological associations and/or issues with parameter identifiability.

```r
plot_pairs(model_output, type = "biphasic")
```

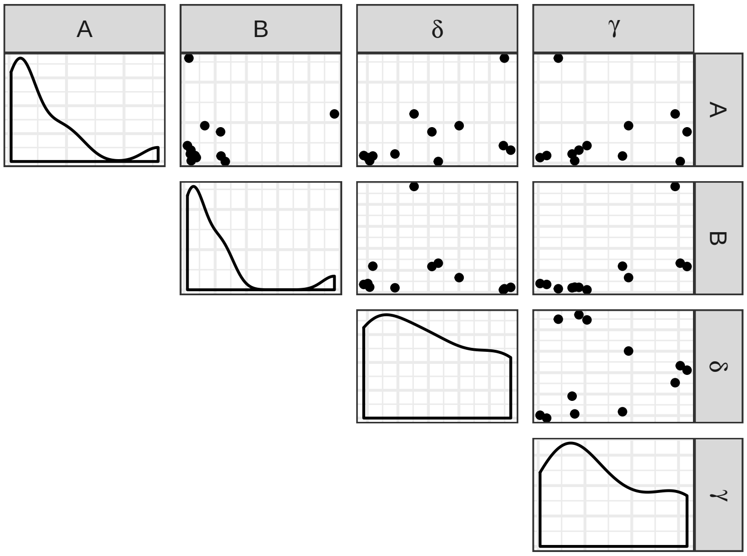


```r
plot_pairs(model_output, type = "single")
```

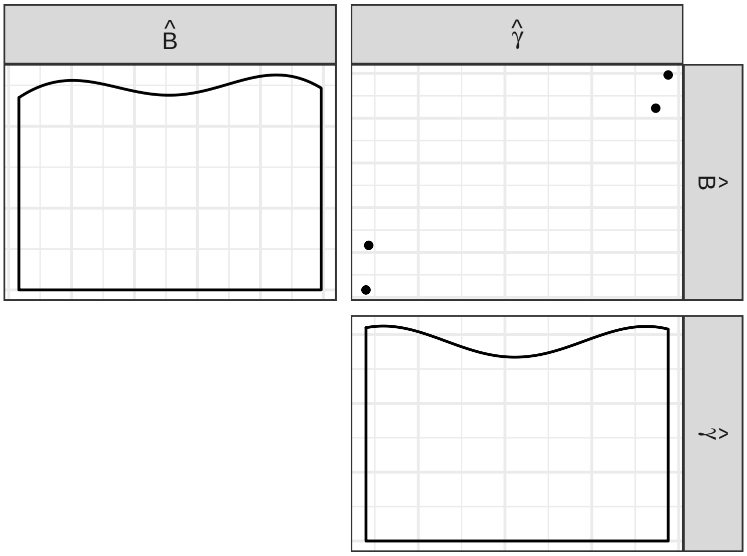


## Time to suppression

In addition to fitting the biphasic and single phase models, we can calculate the time to viral suppression (TTS) using both the parametric and non-parameteric methods (see main text for more details). Here we set the suppression threshold to be the same as the detection threshold (i.e. we want to know when viral load drops below the detection threshold of the assay). First, to get parametric estimates from the model output, we use `get_TTS()` with the argument `parametric = TRUE`. We can subsequently obtain median and SD statistics, and the total number of subjects included in the analysis, using the `summarize()` function from `dplyr`.

```r
TTSparametric <- get_TTS(model_output = model_output, parametric = TRUE,
                         suppression_threshold = 100)
head(TTSparametric)
```

```
## # A tibble: 6 x 4
##      id   TTS model    calculation
##   <dbl> <dbl> <chr>    <chr>
## 1     1  69.2 biphasic parametric
## 2    13  48.7 biphasic parametric
## 3    17  73.1 biphasic parametric
## 4    19  65.0 biphasic parametric
## 5    26 122.  biphasic parametric
## 6    27  69.8 biphasic parametric
```

```
TTSparametric %>% summarize(median = median(TTS), SD = sd(TTS), N = n())
```

```
## # A tibble: 1 x 3
##   median    SD     N
##    <dbl> <dbl> <int>
## 1   65.7  31.0    16
```

Alternatively, to calculate non-parametric TTS estimates, we set the argument `parametric = FALSE`, and supply the original data using `data = actg315`, rather than the model output. The estimates are similar to those for the parametric method. However, given the less stringent conditions for inclusion in the non-parametric analysis (there is no minimum requirement on the number of observations), we are able to estimate TTS for one additional subject.

```
TTSnonparametric <- get_TTS(data = actg315, parametric = FALSE,
                                suppression_threshold = 100)
head(TTSnonparametric)
```

```
## # A tibble: 6 x 3
##      id   TTS calculation
##   <dbl> <dbl> <chr>
## 1     1 69.8  non-parametric
## 2     2  6.89 non-parametric
## 3     4 43.3  non-parametric
## 4     5 27.7  non-parametric
## 5    13 54.0  non-parametric
## 6    17 77.9  non-parametric
```

```
TTSnonparametric %>% summarize(median = median(TTS), SD = sd(TTS), N = n())
```

```
## # A tibble: 1 x 3
##   median    SD     N
##    <dbl> <dbl> <int>
## 1   69.8  37.9    17
```

We can also plot the histograms for both methods using `plot_TTS()`.

```
plot_TTS(TTSparametric, bins = 6, textsize = 7)
```

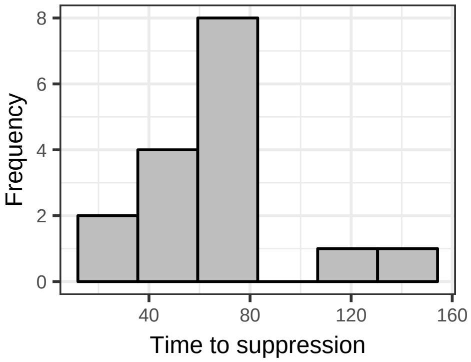


```r
plot_TTS(TTSnonparametric, bins = 6, textsize = 7)
```

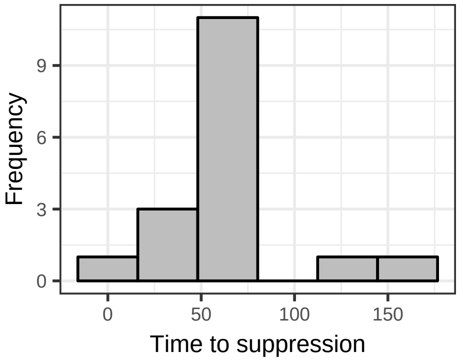


## References for the ACTG 315 data

1. Lederman, M.M., Connick, E., Landay, A., Kuritzkes, D.R., Spritzler, J., St. Clair, M., Kotzin, B.L., Fox, L., Heath Chiozzi, M., Leonard, J.M., Rousseau, F., Wade, M., Roe, J.D., Martinez, A., Kessler, H.: Immunologic Responses Associated with 12 Weeks of Combination Antiretroviral Therapy Consisting of Zidovudine, Lamivudine, and Ritonavir: Results of AIDS Clinical Trials Group Protocol 315. Journal of Infectious Diseases 178(1), 70–79 (1998). doi:10.1086/515591

2. Wu, H., Ding, A.A.: Population HIV-1 dynamics in vivo: applicable models and inferential tools for virological data from AIDS clinical trials. Biometrics 55(2), 410–418 (1999)

3. Connick, E., Lederman, M., Kotzin, B., Spritzler, J., Kuritzkes, D., St. Clair, M., Sevin, A., Fox, L., Chiozzi, M., Leonard, J., Rousseau, F., D'Arc Roe, J., Martinez, A., Kessler, H., Landay, A.: Immune Reconstitution in the First Year of Potent Antiretroviral Therapy and Its Relationship to Virologic Response. The Journal of Infectious Diseases 181(1), 358–363 (2000). doi:10.1086/315171
