# Supplementary material for: ushr: Understanding suppression of HIV in R
Source: BMC Bioinformatics. 2020 Feb 11;21:52. doi: 10.1186/s12859-020-3389-x (PMC7014720; doi:10.1186/s12859-020-3389-x)

Additional File 3: Parameter estimates are accurate for sufficiently high resolution data

Sinead E. Morris, Luise Dziobek-Garrett, Andrew J. Yates

This file includes all code used to generate simulated from the main text and analyze it using `ushr`.

Basic setup

First we load all required packages and define baseline plot settings. Note the package `parallel` enables the algorithms to be run in parallel, and the packages `cowplot` and `viridis` are used during plot creation.

```
require(ushr)
require(parallel)
require(cowplot)
require(viridis)

basetext <- 10
basepoint <- 2
baseline <- 1

mytheme <- theme_bw() + theme(axis.text = element_text(size = basetext),
                              axis.title = element_text(size = basetext + 1),
                              legend.text = element_text(size = basetext),
                              legend.title = element_text(size = basetext + 1),
                              strip.text.x = element_text(size = basetext + 1))
```

Simulation function

Next we define the `simulate_studies()` function that combines the following steps:

1. simulating the data (`simulate_data()`), collecting the original parameter estimates, and calculating the true TTS values (`get_parametricTTS()`);
2. fitting the model to the simulated data (`ushr()`) and getting the corresponding TTS (`get_TTS()`) and parameter estimates (`biphasicCI`);
3. getting true parameter estimates from just the subset fit with the biphasic model (`true`);
4. calculating the average deviation scores.

Note the functions `simulate_data()`, `get_parametricTTS()`, `ushr()`, and `get_TTS()` are all from `ushr`.

```
simulate_studies <- function(simulation, nsubjects, detection_threshold, mean_params){

    # 1. simulate data for 3 resolutions: low, intermediate, and high
    data_low <- simulate_data(nsubjects = nsubjects,
                              detection_threshold = detection_threshold,
                             min_datapoints = 3, max_datapoints = 12,
                             mean_params = mean_params) %>%
```

```r
    mutate(type = "low")

data_int <- simulate_data(nsubjects = nsubjects,
                        detection_threshold = detection_threshold,
                        min_datapoints = 6, max_datapoints = 24,
                        mean_params = mean_params) %>%
    mutate(type = "intermediate")

data_high <- simulate_data(nsubjects = nsubjects,
                            detection_threshold = detection_threshold,
                           min_datapoints = 12, max_datapoints = 48,
                           mean_params = mean_params) %>%
    mutate(type = "high")

 # Get true parameter values and calculate TTS
data_params <- rbind(data_low, data_int, data_high) %>%
    mutate(simulation = simulation) %>%
    distinct(id, A, delta, B, gamma, type, simulation) %>%
    mutate(shortlifespan = 1/delta, longlifespan = 1/gamma,
           logA = log10(A), logB = log10(B)) %>%
    mutate(TTS = get_parametricTTS(params = ., rootfunction = biphasic_root,
                                   suppression_threshold = detection_threshold,
                                   uppertime = 365))

data_all <- rbind(data_low, data_int, data_high) %>%
    mutate(simulation = simulation)

# 2. Fit model to each dataset
model_low <- ushr(data = data_low, detection_threshold = detection_threshold)

model_int <- ushr(data = data_int, detection_threshold = detection_threshold)

model_high <- ushr(data = data_high, detection_threshold = detection_threshold)

# Get TTS estimates
TTS_low <- get_TTS(model_output = model_low,
                   suppression_threshold = detection_threshold, parametric = TRUE) %>%
    filter(model == "biphasic") %>%
    select(id, TTS) %>% mutate(type = "low")

TTS_int <- get_TTS(model_output = model_int,
                 suppression_threshold = detection_threshold, parametric = TRUE) %>%
    filter(model == "biphasic") %>%
    select(id, TTS) %>% mutate(type = "intermediate")

TTS_high <- get_TTS(model_output = model_high,
                    suppression_threshold = detection_threshold, parametric = TRUE) %>%
    filter(model == "biphasic") %>%
    select(id, TTS) %>% mutate(type = "high")

TTS_all <- rbind(TTS_low, TTS_int, TTS_high)
```

```r
# Get parameters from biphasic model
biphasic_low <- model_low$biphasicCI %>% mutate(type = "low")

biphasic_int <- model_int$biphasicCI %>% mutate(type = "intermediate")

biphasic_high <- model_high$biphasicCI %>% mutate(type = "high")

biphasic_all <- rbind(biphasic_low, biphasic_int, biphasic_high) %>%
    mutate(simulation = simulation) %>%
    distinct(id, param, estimate, type, simulation) %>%
    spread(param, estimate) %>%
    mutate(shortlifespan = 1/delta, longlifespan = 1/gamma,
           logA = log10(A), logB = log10(B)) %>%
    left_join(TTS_all) %>%
    gather(param, estimate, A:logB, TTS)


# 3. Get true parameter values for subjects fit with the biphasic model
true_low <-  data_low %>% filter(id %in% biphasic_low$id) %>%
    distinct(id, A, delta, B, gamma, type) %>%
    mutate(TTS = get_parametricTTS(params = ., rootfunction = biphasic_root,
                                   suppression_threshold = detection_threshold,
                                   uppertime = 365))

true_int <- data_int %>% filter(id %in% biphasic_int$id) %>%
    distinct(id, A, delta, B, gamma, type) %>%
    mutate(TTS = get_parametricTTS(params = ., rootfunction = biphasic_root,
                                   suppression_threshold = detection_threshold,
                                   uppertime = 365))

true_high <- data_high %>% filter(id %in% biphasic_high$id) %>%
    distinct(id, A, delta, B, gamma, type) %>%
    mutate(TTS = get_parametricTTS(params = ., rootfunction = biphasic_root,
                                   suppression_threshold = detection_threshold,
                                   uppertime = 365))

true_all <- rbind(true_low, true_int, true_high) %>%
    mutate(shortlifespan = 1/delta, longlifespan = 1/gamma,
           logA = log10(A), logB = log10(B)) %>%
    gather(param, true, A:gamma, shortlifespan:logB, TTS) %>%
    mutate(simulation = simulation)


# 4. Get deviation scores
stats <- biphasic_all  %>%
    left_join(true_all) %>%
    group_by(type, param) %>% mutate(n = n()) %>%
    mutate(deviation = (true - estimate)/true) %>%
    summarize(deviation = sum(deviation)/unique(n)) %>% ungroup() %>%
    arrange(desc(abs(deviation))) %>% mutate(simulation = simulation)

return(list(data = data_all, data_params = data_params,
            biphasic_all = biphasic_all, stats = stats))
```

```
}
```

Simulate data, fit model, and collect output

We then define the settings for the simulation conditions i.e. detection threshold, number of subjects, and number of repetitions. Then we define the range of mean parameter values from which to sample on each repetition, and generate a matrix of new parameter sets by sampling from this range.

```
set.seed(1234567)

detection_threshold <- 100

nsubjects <- 200
nreps <- 10

param_max <- c(A = 2e5, delta = 0.5, B = 1e4, gamma = 0.05)
param_min <- c(A = 5e4, delta = 0.2, B = 5e3, gamma = 0.03)

paramset <- runif(n = length(param_max) * nreps,
                  min = param_min, max = param_max) %>%
    matrix(., byrow = TRUE, nrow = nreps)

colnames(paramset) <- names(param_max)
```

Once we have defined our simulation conditions, we then run the `simulate_studies()` function for each repetition. Note that we run in parallel using `mclapply()` from `parallel`; the number of cores can be changed according to user preference and machine capability. The analysis takes some time, so we specified `nreps = 10` above as an example only. To recreate the analysis presented in the main text, one must set `nreps = 100`.

```
ncores <- 2

# Run the main function
output <- mclapply(1:nreps, mc.cores = ncores,
                   function(i) simulate_studies(simulation = i, nsubjects,
                                                detection_threshold,
                                                mean_params = paramset[i,]))

# Format output list elements as separate data frames
data_params <- lapply(output, function(x) x$data_params) %>% bind_rows()
data_all <- lapply(output, function(x) x$data_all) %>% bind_rows()

stats <- lapply(output, function(x) x$stats) %>% bind_rows()
biphasic <- lapply(output, function(x) x$biphasic_all) %>% bind_rows()
```

Process and plot output

Fig 4: plot deviation scores

To visualize the average deviation values for each parameter and study, we first collect the true mean values used to simulate each dataset and calculate the associated TTS estimates.

```r
paramdat <- cbind(paramset, simulation = 1:nreps) %>% tbl_df() %>%
    mutate(shortlifespan = 1/delta, longlifespan = 1/gamma,
           logA = log10(A), logB = log10(B)) %>%
        mutate(TTS = get_parametricTTS(params = ., rootfunction = biphasic_root,
                                        suppression_threshold = detection_threshold,
                                        uppertime = 365),
               model = "biphasic", calculation = "parametric") %>%
    gather(param, meanvalue, A:gamma, shortlifespan:logB, TTS)
```

We then plot these true values against the average deviation statistics. From Fig 4 (main text), we can see that estimates of the short-lived lifespan from the low resolution study design consistently have the largest deviation from their true value. Moreover, estimates are more likely to be overestimated (i.e. have a negative deviance) for the shortest of these lifespans. Further details can be found in the main text.

```r
stats %>% left_join(paramdat) %>%
    filter(param %in% c("logA", "logB", "shortlifespan", "longlifespan", "TTS")) %>%
    ggplot() +
    geom_point(aes(x = deviation, y = meanvalue, colour = type), size = 2, alpha = 0.5) +
    facet_wrap(~ param, scales = "free_y", ncol = 2) + mytheme  +
    scale_colour_discrete("Resolution") +
    theme(legend.position = c(0.8, 0.15)) + ylab("True mean parameter value") +
    scale_x_continuous("Average deviation from true value")
```

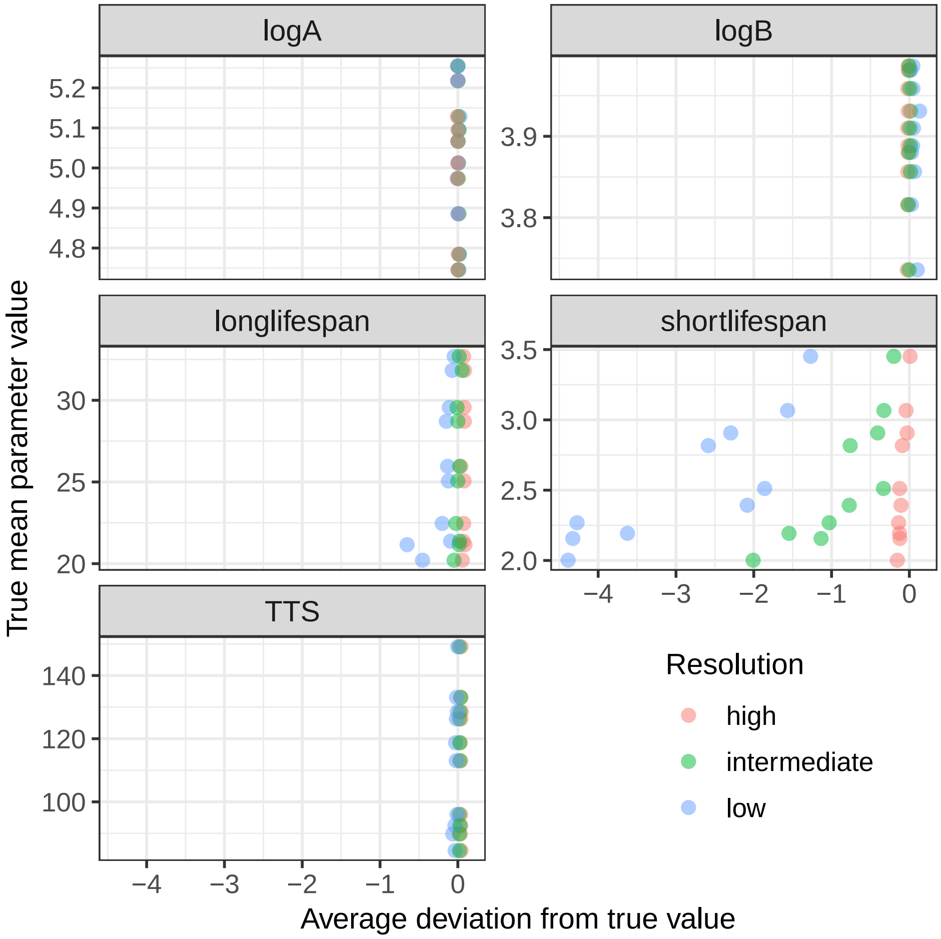

Fig 5: compare estimates with population values

To compare model estimates with the entire study population, we first calculate the median parameter values for each original data set. We do the same for each corresponding subset fit using the biphasic model. Note that we also calculate the number of subjects fit in each dataset (this forms the colour scale in Fig 5).

```
# original values
allvalues <- data_params %>% gather(param, true, A:gamma, shortlifespan:logB, TTS) %>%
    group_by(simulation, type, param) %>% summarize(true = median(true)) %>%
    ungroup()

# number of subjects
nfits <- biphasic %>%
    distinct(id, type, simulation) %>%
    group_by(type, simulation) %>% mutate(nfits = n()) %>% ungroup()

# subset fit with biphasic model
compare_data <- biphasic %>%
    group_by(simulation, type, param) %>% summarize(estimate = median(estimate)) %>%
    ungroup() %>% left_join(allvalues) %>%
    left_join(nfits) %>%
    filter(param %in% c("shortlifespan", "longlifespan", "TTS")) %>%
    distinct(param, estimate, true, simulation, nfits, type)
```

Finally, we plot the true median values against the corresponding estimates from the fitted subset. Note that `plot_grid()` is from the `cowplot` package, and `scale_colour_viridis()` is from the `viridis` package. From Fig 5 (main text) we can see that, in general, estimates from the low resolution studies were the poorest representation of the true population-wide values. In contrast, the intermediate and high resolution studies were in good agreement with the true population average. Further details can be found in the main text.

```
# dummy data to improve clarity of display axes
dummy_long <- data.frame(type = rep(c("high", "intermediate", "low"), each = 2),
                          true = c(1, 35, 1, 35, 1, 35),
                         estimate = c(1, 35, 1, 35, 1, 35))

dummy_short <- data.frame(type = rep(c("high", "intermediate", "low"), each = 2),
                          true = c(1, 20, 1, 20, 1, 20),
                          estimate = c(1, 30, 1, 30, 1, 30))


longplt <- compare_data %>% filter(param == "longlifespan") %>%
    ggplot() +
    geom_point(aes(x = true, y = estimate, colour = nfits),
                              size = 3, alpha = 0.5) +
    geom_abline(aes(intercept = 0, slope = 1), linetype = "dashed") +
    geom_blank(data = dummy_long, aes(x = true, y = estimate)) +
    facet_wrap(~ type, ncol = 1) + mytheme +
    ylab("Estimate (fitted subset)") + xlab("True (all subjects)") +
    scale_colour_viridis("Subset \nsize")

shortplt <- compare_data %>% filter(param == "shortlifespan") %>%
    ggplot() +
    geom_point(aes(x = true, y = estimate, colour = nfits),
                              size = 3, alpha = 0.5) +
    geom_abline(aes(intercept = 0, slope = 1), linetype = "dashed") +
```

```r
    geom_blank(data = dummy_short, aes(x = true, y = estimate)) +
    facet_wrap(~ type, ncol = 1) + mytheme +
    ylab("Estimate (fitted subset)") + xlab("True (all subjects)") +
    scale_colour_viridis(guide = FALSE)


plot_grid(shortplt, longplt, labels = c("A", "B"),
          label_size = 14, rel_widths = c(1, 1.4), nrow = 1)
```

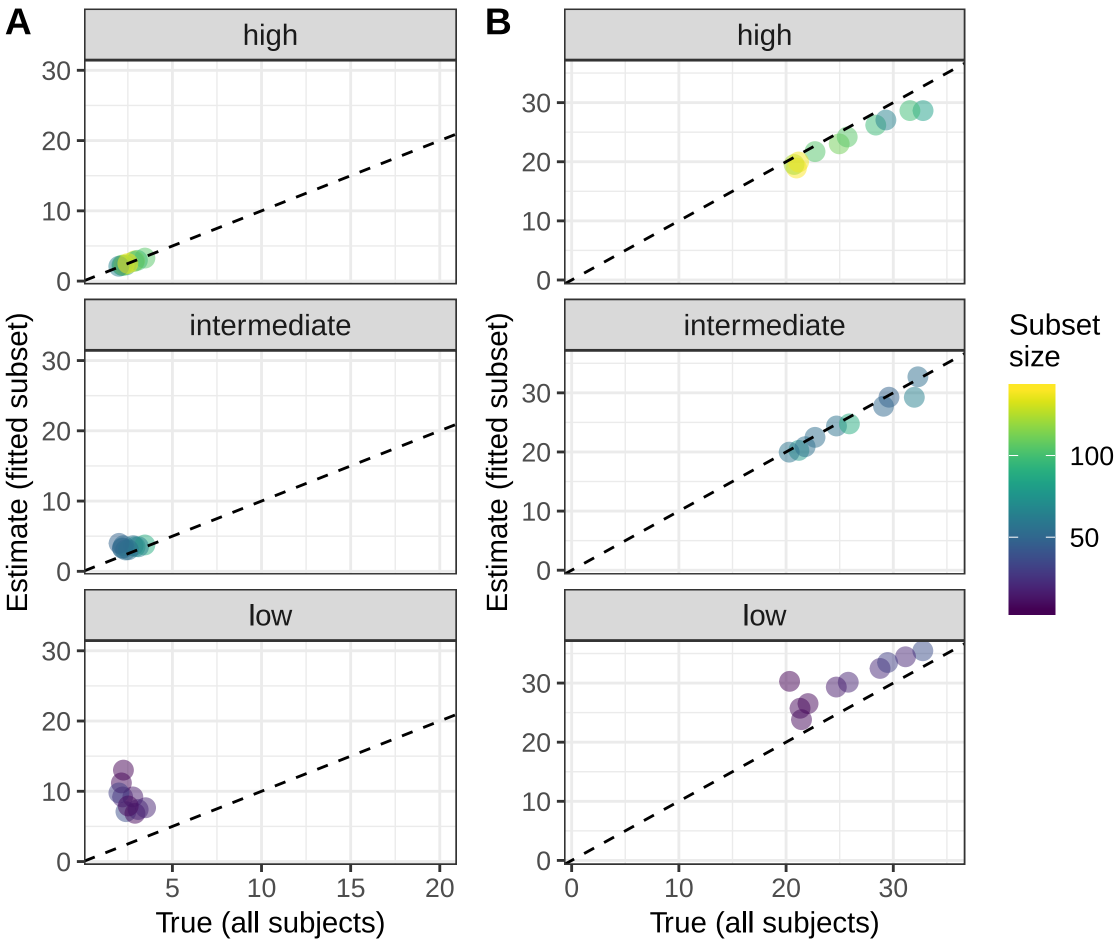

Supplement: Supplementary file 3 — Additional file 3 Code used to generate all simulated data and conduct corresponding ushr analyses. [file 12859_2020_3389_MOESM3_ESM.pdf]
