# Supplementary material for: ushr: Understanding suppression of HIV in R
Source: BMC Bioinformatics. 2020 Feb 11;21:52. doi: 10.1186/s12859-020-3389-x (PMC7014720; doi:10.1186/s12859-020-3389-x)
Supplement: Supplementary file 4 — Additional file 4 Code used to compare ushr analyses with nonlinear mixed effects modeling at the population level. [file 12859_2020_3389_MOESM4_ESM.pdf]

Additional File 4: ushr performs comparably to nonlinear mixed effects modeling

Sinead E. Morris, Luise Dziobek-Garrett, Andrew J. Yates

This file includes all code used to compare `ushr` analysis of simulated data with nonlinear effects modeling (NLME).

Basic setup

First we load all required packages and then define the baseline plot settings. Note that we perform nonlinear mixed effects modeling using the `saemix` package; we run our algorithms in parallel using the `parallel` package; and we use the `cowplot` package during plot creation.

```
require(ushr)
require(saemix)
require(parallel)
require(cowplot)

basetext <- 10
basepoint <- 2
baseline <- 1

mytheme <- theme_bw() + theme(axis.text = element_text(size = basetext),
                              axis.title = element_text(size = basetext + 1),
                              legend.text = element_text(size = basetext),
                              legend.title = element_blank(),
                              strip.text.x = element_text(size = basetext + 1))
```

NLME fitting function

Next we define the functions needed to fit the NLME model using `saemix`:

1. `get_model()` specifies the biphasic formula to be fit by `saemix`; this is required input that must be specified by the user.
2. `do_filter()` filters the raw data so that only individuals that meet our criteria are included in the fitting procedure; this differs from the filtering procedure used by `ushr` in that there is no requirement on the minimum number of observations.
3. `fit_saemix` fits the biphasic function to the filtered data for a specific repetition and resolution type.
4. `fit_all` applies `fit_saemix` to all resolutions.

```
# 1. define the model formula
get_model <- function(psi, id, x){
  A <- psi[id,1]
  B <- psi[id,2]
  delta <- psi[id,3]
  gamma<- psi[id,4]
  t <- x[,1]

  V <- A * exp( - delta * t) + B * exp ( - gamma * t)
  return(V)
```

```
}


# 2. filter raw data
do_filter <- function(data, detection_threshold = 100,
                      censortime = 365, decline_buffer = 500,
                      threshold_buffer = 10){

    filtered <- data %>%
        mutate(vl = case_when(vl <= detection_threshold ~ detection_threshold/2,
                              vl >= detection_threshold ~ vl) ) %>%
        # Look at only those who reach control within user defined censortime
        filter(time <= censortime) %>% group_by(id, simulation, type) %>%
        filter(any(vl <= detection_threshold)) %>% ungroup() %>%
        # Isolate data from the highest VL measurement (in points 1-3; same as ushr default)
        # to the first point below detection
        filter(!is.na(vl)) %>% group_by(id, simulation, type) %>%
        slice(which.max(vl[1:3]):Position(function(x) x <= detection_threshold, vl)) %>%
        ungroup() %>%
        # Only keep VL sequences that are decreasing with user defined buffer
        group_by(id, simulation, type) %>%
        filter(all(vl <= cummin(vl) + decline_buffer)) %>%
        group_by(id, simulation, type) %>%
        mutate(n = n(), index = 1:n(),
               tag = ifelse(vl[n-1] - vl[n] < threshold_buffer, TRUE, FALSE) ) %>%
        filter(!(tag == TRUE & index == n)) %>%
        ungroup() %>% select(-index, -n, -tag)

    return(filtered)

}

# 3. fit the biphasic function to the filtered data
fit_saemix <- function(data0, model){
    data00 <<- data0

    saemix_data <- saemixData(name.data = data00, name.group = "id",
                              name.predictors = "time", name.response = "vl",
                              verbose = FALSE)

    # define starting guess (same as the default guess in ushr )
    guess0 <- c(A = 10000, delta = 0.68, B = 1000, gamma = 0.03)

    # construct model
    saemix_model <- saemixModel(model = model,
                                psi0 = matrix(guess0, ncol = length(guess0), byrow = TRUE,
                                              dimnames = list(NULL, c("A", "B", "delta", "gamma"))),
                                error.model = "exponential", transform.par = c(1,1,1,1),
                                verbose = FALSE)

    # set fitting options and fit model
    saemix.options <- list(map = TRUE, fim = TRUE, ll.is = FALSE,
                           displayProgress = FALSE, seed = 1234567,
```

```
                              print = FALSE, warnings = FALSE,
                              save = FALSE, save.graphs = FALSE)
    saemix_fit <- saemix(saemix_model, saemix_data, saemix.options)

    # get population-level parameters
    poppar <- coef(saemix_fit)$fixed

    poppar <- data.frame(param = names(poppar),
                         estimate = signif(poppar, 3),
                         row.names = NULL,stringsAsFactors = FALSE )  %>%
                rbind(., c("1/delta", signif(1/as.numeric(.[3, 2]),3)),
                          c("1/gamma", signif(1/as.numeric(.[4, 2]),3) ) ) %>%
                mutate(simulation = data0$simulation[1], type = data0$type[1])

    return(poppar)
}

# 4. apply to all resolution types (low, intermediate, high)
fit_all <- function(i, tmpdata, model){

    tmp <- list()
    types <- unique(tmpdata$type)

    for (j in 1:length(types)) {
        data0 <- tmpdata %>% filter(simulation == i, type == types[j])

        tmp[[j]] <- fit_saemix(data0 = data0, model = model)
    }

    pop <- bind_rows(tmp)
    return(pop)
}
```

Load simulated data, fit NLME model, and collect output

To run the analysis, we first load the simulated data (`AdditionalFile6.RData`), true parameter values (`AdditionalFile7.RData`), and corresponding parameter estimates (`AdditionalFile8.RData`) from the `ushr` analysis. These can also be generated using Additional File 3 (AF3) with `nreps = 100`. We then filter the simulated data to identify individuals that adhere to our inclusion criteria.

```
load("AdditionalFile6.RData")      # load 'data_all' data frame from AF3
load("AdditionalFile7.RData")      # load 'data_params' data frame from AF3
load("AdditionalFile8.RData")      # load 'biphasic' data frame from AF3

filtered <- do_filter(data_all)
```

We then run the `fit_all()` function for each parameter repetition. Note that we run in parallel using `mclapply()` from `parallel`; the number of cores can be changed according to user preference and machine capability. The analysis takes some time, so we specify `nreps = 10` as an example only. To recreate the analysis presented in the main text, one can use the commented out code that covers all repetitions present in the filtered data.

```
nreps <- 10
```

```r
#nreps <- max(filtered$simulation)

ncores <- 2

output <- mclapply(1:nreps, mc.cores = ncores,
                   function(i) fit_all(i,
                                       tmpdata = filtered,
                                       model = get_model))

# convert output to data frame
popCI <- output %>% bind_rows()
```

Process and plot output

Fig 6: plot deviation scores

To compare `ushr` with the NLME approach, we first collect the true mean parameter values used to simulate each original dataset. We then merge this with the corresponding population-level parameter estimates from `saemix`.

```r
# true values
allvals <- data_params %>%
    gather(param, true, A:gamma, shortlifespan:logB) %>%
    group_by(simulation, type, param) %>%
    summarize(true = median(true)) %>%
    ungroup()

# saemix estimates
saemixvals <- popCI %>%
    mutate(estimate = as.numeric(estimate),
           param = ifelse(param == "1/delta", "shortlifespan", param),
           param = ifelse(param == "1/gamma", "longlifespan", param)) %>%
    # merge with true values
    left_join(allvals) %>% mutate(analysis = "NLME")
```

We also collect the estimates obtained from `ushr`, and then merge these with the true values and `saemix` estimates.

```r
# ushr estimates
compare_data <- biphasic %>% group_by(simulation, type, param) %>%
    summarize(estimate = median(estimate)) %>%
    ungroup() %>% left_join(allvals) %>%
    filter(param %in% c("shortlifespan", "longlifespan")) %>%
    distinct(param, estimate, true, simulation, type) %>%
    mutate(analysis = "individual") %>%
    select(param, estimate, true, simulation, type, analysis)

# merge all together
compareAll <- saemixvals %>%
    select(param, estimate, true, simulation, type, analysis) %>%
    filter(param %in% c("shortlifespan", "longlifespan")) %>%
    rbind(.,compare_data) %>%
    distinct(param, estimate, true, simulation, type, analysis)
```

From Fig 6 (main text) we can see that estimates from the NLME approach are similar to, or marginally worse than, those obtained from `ushr` for the intermediate and high resolution data. In contrast, the NLME lifespans were closer to the true population average for the low resolution data. Further details can be found in the main text.

```
# dummy data to improve clarity of display axes
dummy_long <- data.frame(type = rep(c("high", "intermediate", "low"), each = 4),
                         true = rep(c(18, 75, 18, 75, 18, 75), each = 2),
                         estimate = rep(c(18, 75, 18, 75, 18, 75), each = 2),
                         analysis = rep(c("NLME", "ODE"), times = 6) )

dummy_short <- data.frame(type = rep(c("high", "intermediate", "low"), each = 4),
                          true = rep(c(1, 15, 1, 15, 1, 15), each = 2),
                          estimate = rep(c(1, 15, 1, 15, 1, 15), each = 2),
                          analysis = rep(c("NLME", "ODE"), times = 6) )


# Long lifespan plot
compareLong <- compareAll %>% filter(param == "longlifespan") %>%
    ggplot() +
    geom_point(aes(x = true, y = estimate, colour = analysis),
                           size = 3, alpha = 0.2) +
    geom_abline(aes(intercept = 0, slope = 1), linetype = "dashed") +
    geom_blank(data = dummy_long, aes(x = true, y = estimate)) +
    facet_wrap(~ type, ncol = 1) + mytheme +
    ylab("Estimate (fitted subset)") + xlab("True (all subjects)") +
    scale_colour_discrete(name = NULL) +
    theme(legend.position = c(0.79, 0.82)) +
    guides(color = guide_legend(override.aes = list(alpha = 1)))

# Short lifespan plot
compareShort <- compareAll %>% filter(param == "shortlifespan") %>%
    ggplot() +
    geom_point(aes(x = true, y = estimate, colour = analysis),
                           size = 3, alpha = 0.2) +
    geom_abline(aes(intercept = 0, slope = 1), linetype = "dashed") +
    geom_blank(data = dummy_short, aes(x = true, y = estimate)) +
    facet_wrap(~ type, ncol = 1) + mytheme +
    ylab("Estimate (fitted subset)") + xlab("True (all subjects)") +
    scale_colour_discrete(guide = FALSE)

# Combine plots using plot_grid() from cowplot package
print(
    plot_grid(compareShort, compareLong,
              labels = c("A", "B"), label_size = 14)
)
```

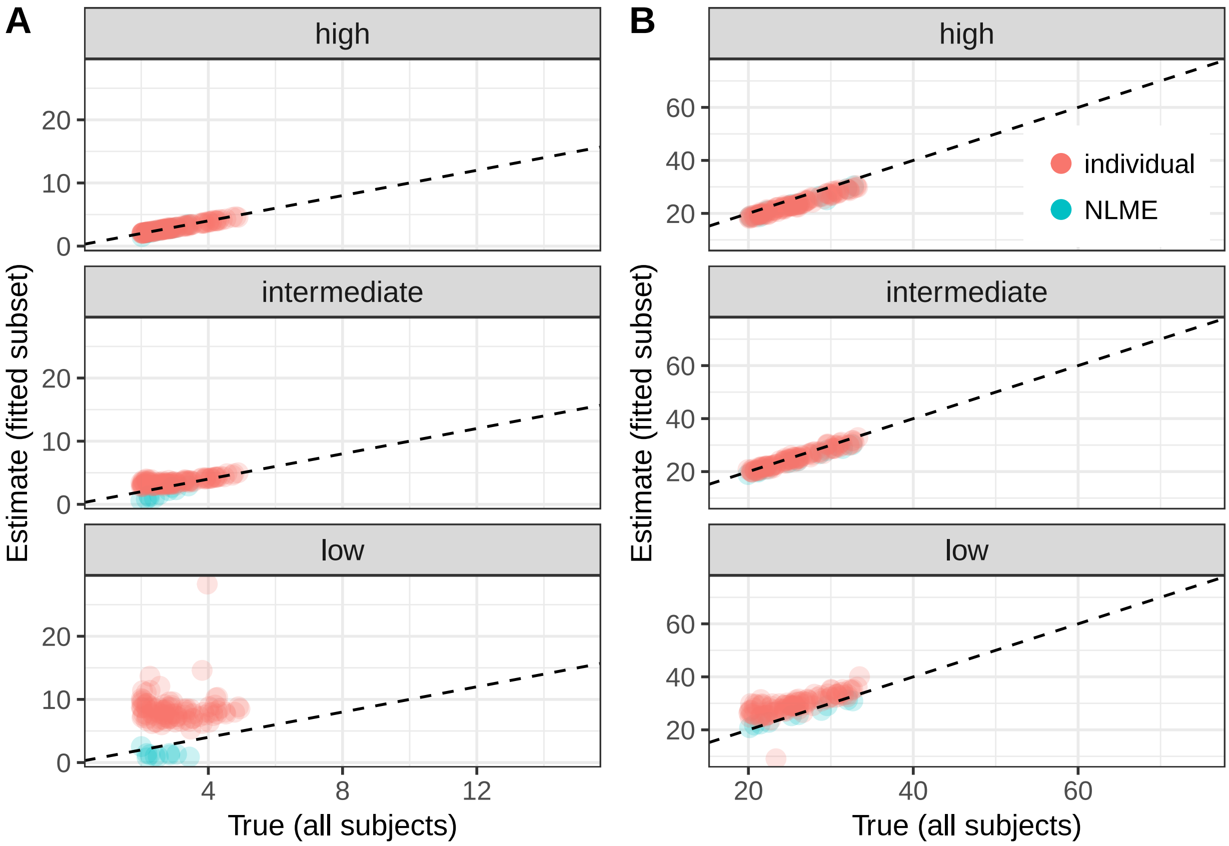

A
B
high
intermediate
low
Estimate (fitted subset)
True (all subjects)
individual
NLME
0
10
20
4
8
12
20
40
60
