# Supplementary material for: ushr: Understanding suppression of HIV in R
Source: BMC Bioinformatics. 2020 Feb 11;21:52. doi: 10.1186/s12859-020-3389-x (PMC7014720; doi:10.1186/s12859-020-3389-x)
Supplement: Supplementary file 5 — Additional file 5 Additional individuals fit using nonlinear mixed effects modeling. [file 12859_2020_3389_MOESM5_ESM.pdf]

Additional File 5: Individuals fit using only nonlinear mixed effects modeling

Sinead E. Morris, Luise Dziobek-Garrett, Andrew J. Yates

Nonlinear mixed effects modeling (NLME) enables individuals with sparse data to be included in model fitting i.e. unlike `ushr` there is no minimum requirement on the number of observations. This file includes code used to investigate individuals who were included in the NLME analysis, but were not fit using `ushr`.

Basic setup

First we load all required packages and define baseline plot settings. Note that we perform nonlinear mixed effects modeling using the `saemix` package, and we run our algorithms in parallel using the `parallel` package.

```
require(ushr)
require(saemix)
require(parallel)
require(cowplot)

basetext <- 10
basepoint <- 2
baseline <- 1

mytheme <- theme_bw() + theme(axis.text = element_text(size = basetext),
                              axis.title = element_text(size = basetext + 1),
                              legend.text = element_text(size = basetext),
                              legend.title = element_blank(),
                              strip.text.x = element_text(size = basetext + 1))
```

NLME fitting function

Next we define the functions needed to fit the NLME model using `saemix`.

1. `get_model()` specifies the biphasic formula to be fit by `saemix`; this is required input that must be specified by the user.
2. `do_filter()` filters the raw data so that only individuals that meet our criteria are included in the fitting procedure; this differs from the filtering procedure used by `ushr` in that there is no requirement on the minimum number of observations.
3. `fit_saemix` fits the biphasic function to the filtered data for a specific repetition and resolution type and returns the individual fits.
4. `fit_all` applies `fit_saemix` to all resolutions.

```
# 1. define the model formula
get_model <- function(psi, id, x){
  A <- psi[id,1]
  B <- psi[id,2]
  delta <- psi[id,3]
```

```
  gamma<- psi[id,4]
  t <- x[,1]

  V <- A * exp( - delta * t) + B * exp ( - gamma * t)
  return(V)
}


# 2. filter raw data
do_filter <- function(data, detection_threshold = 100,
                      censortime = 365, decline_buffer = 500,
                      threshold_buffer = 10){

    filtered <- data %>%
        mutate(vl = case_when(vl <= detection_threshold ~ detection_threshold/2,
                              vl >= detection_threshold ~ vl) ) %>%
        # Look at only those who reach control within user defined censortime
        filter(time <= censortime) %>% group_by(id, simulation, type) %>%
        filter(any(vl <= detection_threshold)) %>% ungroup() %>%
        # Isolate data from the highest VL measurement (in points 1-3; same as ushr default)
        # to the first point below detection
        filter(!is.na(vl)) %>% group_by(id, simulation, type) %>%
        slice(which.max(vl[1:3]):Position(function(x) x <= detection_threshold, vl)) %>%
        ungroup() %>%
        # Only keep VL sequences that are decreasing with user defined buffer
        group_by(id, simulation, type) %>%
        filter(all(vl <= cummin(vl) + decline_buffer)) %>%
        group_by(id, simulation, type) %>%
        mutate(n = n(), index = 1:n(),
               tag = ifelse(vl[n-1] - vl[n] < threshold_buffer, TRUE, FALSE) ) %>%
        filter(!(tag == TRUE & index == n)) %>%
        ungroup() %>% select(-index, -n, -tag)

    return(filtered)

}

# 3. fit the biphasic function to the filtered data and return individual fits
fit_saemix <- function(data0, model){
    data00 <<- data0

    saemix_data <- saemixData(name.data = data00, name.group = "id",
                              name.predictors = "time", name.response = "vl",
                              verbose = FALSE)

    # define starting guess (same as the default guess in ushr )
    guess0 <- c(A = 10000, delta = 0.68, B = 1000, gamma = 0.03)

    # construct model
    saemix_model <- saemixModel(model = model,
                                psi0 = matrix(guess0, ncol = length(guess0), byrow = TRUE,
                                              dimnames = list(NULL, c("A", "B", "delta", "gamma"))),
                                error.model = "exponential", transform.par = c(1,1,1,1),
```

```
                                verbose = FALSE)

    # set fitting options and fit model
    saemix.options <- list(map = TRUE, fim = TRUE, ll.is = FALSE,
                           displayProgress = FALSE, seed = 1234567,
                           print = FALSE, warnings = FALSE,
                           save = FALSE, save.graphs = FALSE)
    saemix_fit <- saemix(saemix_model, saemix_data, saemix.options)

    # get fitted model predictions for each individual
    fits <- saemix.predict(saemix_fit)["results"]["ipred"]

    if (length(fits > 0)){
        fits <- data0 %>% mutate(fit = fits)
    } else{
        fits <- data0 %>% mutate(fit = NA)
    }

    return(fits)
}

# 4. apply to each resolution
fit_all <- function(i, tmpdata, model){

    idlist <- list()
    fitlist <- list()

    res <- c("low", "intermediate", "high")

    for(j in 1:length(res)){
       data0 <- tmpdata %>% filter(simulation == i, type == res[j])

       fitlist[[j]] <- fit_saemix(data0 = data0, model = model)
    }

    fitdat <- fitlist %>% bind_rows()

    return(list(fits = fitdat))
}
```

Load simulated data, fit NLME model, and collect output

To run the analysis, we first load the simulated data (`AdditionalFile6.RData`), true parameter values (`AdditionalFile7.RData`), and corresponding parameter estimates (`AdditionalFile8.RData`) from the `ushr` analysis. These can also be generated using Additional File 3 (AF3) with `nreps = 100`. We then filter the simulated data to identify individuals that adhere to our inclusion criteria.

```
load("AdditionalFile6.RData")       # load 'data_all' data frame from AF3
load("AdditionalFile7.RData")       # load 'data_params' data frame from AF3
load("AdditionalFile8.RData")       # load 'biphasic' data frame from AF3

filtered <- do_filter(data_all)
```

We then run the `fit_all()` function for each parameter repetition. Note that we run in parallel using `mclapply()` from `parallel`; the number of cores can be changed according to user preference and machine capability. The analysis takes some time, so we specify `nreps = 10` for illustrative purposes.

```
nreps <- 10

ncores <- 2

output <- mclapply(1:nreps, mc.cores = ncores,
                   function(i) fit_all(i,
                                       tmpdata = filtered,
                                       model = get_model))

# convert output to data frame
NLMEfits <- lapply(output, function(x) x$fits) %>% bind_rows()
```

Process output

To isolate individuals fit using just the NLME approach, we first find all those fit using the biphasic model in `ushr` (`ushrpars`). We then use the `isolate()` function to remove these from the subset of NLME fitted individuals. Note that individuals who were fit using the single phase model in `ushr` will not be removed from the NLME subset at this stage.

```
ushrpars <- biphasic %>% distinct(id, simulation, type)

isolate <- function(i, NLMEdata, ushrdata){

    NLMEtmp <- list()

    res <- c("low", "intermediate", "high")

    for(j in 1:length(res)){
        ushrtmp <- ushrdata %>% filter(simulation == i, type == res[j])

        NLMEtmp[[j]] <- NLMEdata %>%
            filter(simulation == i, type == res[j], !(id %in% ushrtmp$id))
    }

    NLMEtmp <- NLMEtmp %>% bind_rows()
}

NLMEfits_isolated <- mclapply(1:nreps, mc.cores = ncores,
                   function(i) isolate(i,
                                       NLMEdata = NLMEfits,
                                       ushrdata = ushrpars)) %>% bind_rows()
```

We then calculate the *number* of individuals in each simulation and resolution that were fit using just NLME (`isolate_biphasic`). We also calculate the same quantity whilst accounting for individuals who were fit using the single phase model in ushr (`isolate_single`). This is equivalent to removing all individuals with at least three observations (the minimum number of observations required for inclusion in the single phase fitting). As expected, the number of additional individuals fit with NLME for the biphasic model increases as the resolution decreases. However, the numbers are substantially lower when we also remove individuals fit using the single phase model in `ushr`.

```r
isolate_biphasic <- NLMEfits_isolated %>% group_by(simulation, type, id) %>%
    summarize(n = n()) %>% ungroup() %>%
    group_by(simulation, type) %>% summarize(n = n()) %>%
    ungroup() %>% mutate(sim = "biphasic")

isolate_single <- NLMEfits_isolated %>% group_by(simulation, type, id) %>%
    summarize(n = n()) %>% filter(n < 3) %>% ungroup() %>%
    group_by(simulation, type) %>% summarize(n = n()) %>%
    ungroup() %>% mutate(sim = "single")

rbind(isolate_biphasic, isolate_single) %>%
    ggplot() + geom_boxplot(aes(y = n, x = type, colour = sim), width = 0.05) +
    mytheme + xlab("Resolution" ) + ylab("# additional individuals") +
    scale_colour_discrete(labels = c("biphasic only", "single phase included")) +
    theme(legend.position = c(0.25, 0.85))
```

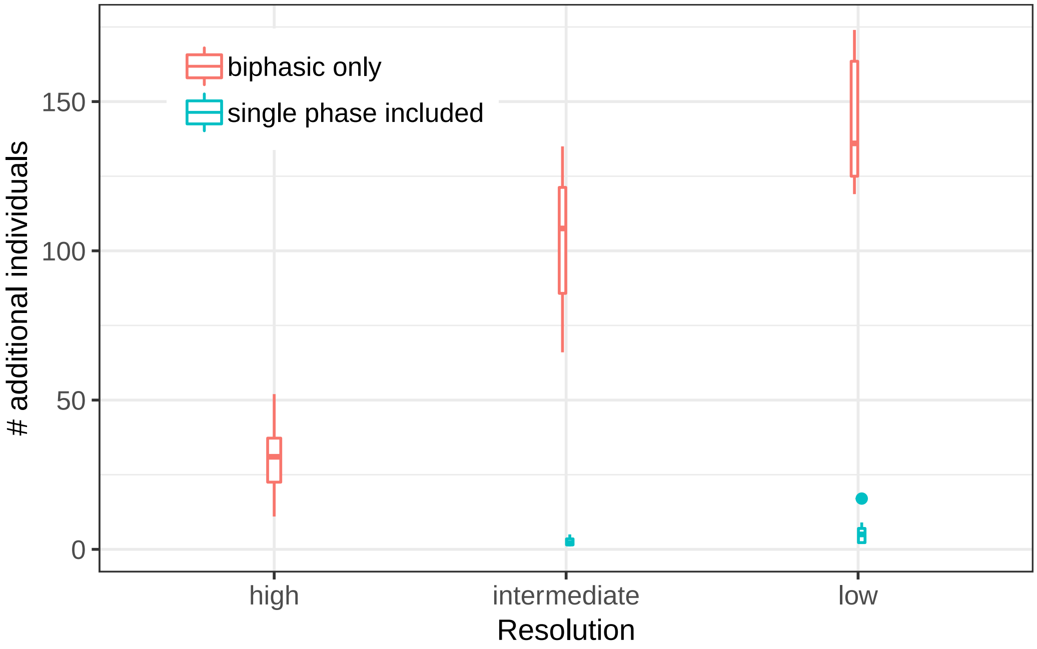

We also plot the individual fits from a randomly sampled, high resolution simulation as a representative example. `ushr` could not fit the biphasic model to these individuals due to insufficient data (e.g. S107), or an undersampled first or second phase (e.g. S30, where the first phase is only captured by one observation). Note that all these individuals could be fit using the single phase model in `ushr`.

```r
set.seed(1234)

NLMEfits_isolated %>% filter(type == "high", simulation == sample(1:nreps)[1]) %>%
    ggplot() + geom_point(aes(x = time, y = vl)) +
    geom_line(aes(x = time, y = fit)) +
    geom_hline(aes(yintercept = 100), linetype = "dashed") +
    facet_wrap(~ id) +
    mytheme + xlab("Time" ) + scale_y_log10("HIV viral load")
```

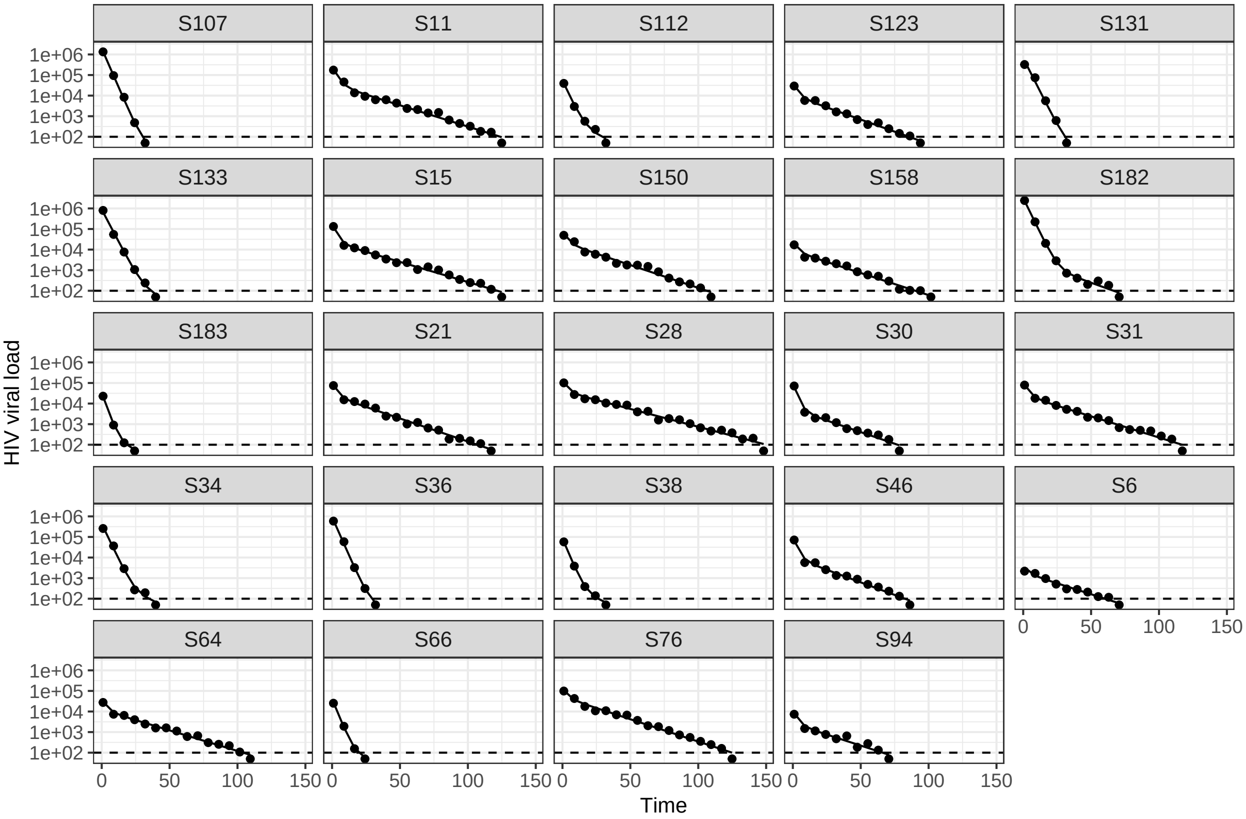
S107
S11
S112
S123
S131
S133
S15
S150
S158
S182
S183
S21
S28
S30
S31
S34
S36
S38
S46
S6
S64
S66
S76
S94
1e+06
1e+05
1e+04
1e+03
1e+02
HIV viral load
0
50
100
150
Time
